# Quality of routine health facility data used for newborn indicators in low- and middle-income countries: A systematic review

**DOI:** 10.7189/jogh.12.04019

**Published:** 2022-04-23

**Authors:** Rebecca Lundin, Ilaria Mariani, Kimberly Peven, Louise T Day, Marzia Lazzerini

**Affiliations:** 1 Institute for Maternal and Child Health – IRCCS “Burlo Garofolo” – WHO Collaborating Centre for Maternal and Child Health, Trieste, Italy; 2 London School of Hygiene & Tropical Medicine, London, UK

## Abstract

**Background:**

High-quality data are fundamental for effective monitoring of newborn morbidity and mortality, particularly in high burden low- and middle-income countries (LMIC).

**Methods:**

We conducted a systematic review on the quality of routine health facility data used for newborn indicators in LMIC, including measures employed. Five databases were searched from inception to February 2021 for relevant observational studies (excluding case-control studies, case series, and case reports) and baseline or control group data from interventional studies, with no language limits. An adapted version (19-point scale) of the Critical Appraisal Tool to assess the Quality of Cross-Sectional Studies (AXIS) was used to assess methodological quality, and results were synthesized using descriptive analysis.

**Results:**

From the 19 572 records retrieved, 34 studies in 16 LMIC countries were included. Methodological quality was high (>14/19) in 32 studies and moderate (10-14/19) in two. Studies were mostly from African (n = 30, 88.2%) and South-East Asian (n = 24, 70.6%) World Health Organization (WHO) regions, with very few from Eastern Mediterranean (n = 2, 5.9%) and Western Pacific (n = 1, 2.9%) ones. We found that only data elements used to calculate neonatal indicators had been assessed, not the indicators themselves. 41 data elements were assessed, most frequently birth outcome. 20 measures of data quality were used, most along three dimensions: 1) completeness and timeliness, 2) internal consistency, and 3) external consistency. Data completeness was very heterogeneous across 26 studies, ranging from 0%-100% in routine facility registers, 0%-100% in patient case notes, and 20%-68% in aggregate reports. One study reported on the timeliness of aggregate reports. Internal consistency ranged from 0% to 96.2% in four studies. External consistency (21 studies) varied widely in measurement and findings, with specificity (6.4%-100%), sensitivity (23.6%-97.6%), and percent agreement (24.6%-99.4%) most frequently reported.

**Conclusions:**

This systematic review highlights a gap in the published literature on the quality of routine LMIC health facility data for newborn indicators. Robust evidence is crucial in driving data quality initiatives at national and international levels. The findings of this review indicate that good quality data collection is achievable even in high-burden LMIC settings, but more efforts are needed to ensure uniformly high data quality for neonatal indicators.

In 2019, UNICEF estimated that 2.4 million babies die globally each year in the first 28 days of life [1]. Additionally, more than 2 million babies die as third-trimester stillbirths. Nearly all (98%) of newborn deaths and stillbirths are in low- and middle-income countries (LMIC) [2,3]. Key global initiatives to reduce neonatal mortality – including the Every Newborn Action Plan (ENAP) [4], the Sustainable Development Goals (SDG) [5], and the Global Strategy for Women’s Children’s and Adolescents’ Health [6] – all note the importance of improving data quality. Improving indicator measurement has advanced progress in other fields, notably human immunodeficiency virus (HIV) treatment and immunization [7].

High-quality data for neonatal health care coverage, content, and quality at health facilities are necessary to support improvements in accountability to accelerate progress towards the reduction of neonatal mortality and morbidity [8-12]. Given that the proportion and the absolute number of hospital births has been increasing globally [13], along with the renewed focus for inpatient care on small or sick newborns, routine facility data are of increasing importance for newborn indicator measurement [14-16].

Poor quality data at facility-level is driven by multiple factors, including excessive and complex reporting systems, lack of standardization and harmonization with reporting systems, lack of digital technology, low health worker motivation, competing demands, lack of feedback, low salaries, poor working conditions, lack of training, and insufficient data management skills [17,18]. Despite efforts to harmonize and standardize routine data collection in LMIC, including the development and implementation of electronic health information systems like District Health Information Software (DHIS2) [19], several studies have identified gaps in the completeness and consistency of facility reporting on maternal and newborn health indicators [20-22]. However, to our knowledge, no published systematic review has documented the quality of newborn data elements used for indicator measurement collected at the facility level in LMIC.

We aimed to systematically review the current evidence regarding quality of newborn indicator measurements, including quality of single data elements (eg, sex, age, mode of birth) in health facility routine data sources in LMICs. We assessed published data on three dimensions of data quality, 1) completeness and timeliness, 2) internal consistency, and 3) external consistency, in three types of facility-level routine data sources: a) individual patient case notes, b) facility registers, and c) aggregate reports, including DHIS or HMIS reports. Individual patient case notes and facility registers correspond to the individual level and aggregate reports to the facility level. We also evaluated measures used to assess quality in included studies.

The results of this review can be used by researchers to expand and further standardize published evidence on the quality of routine health facility data for newborn indicators in LMIC, providing policymakers with the evidence they need to design, target, and evaluate data quality initiatives, ultimately contributing to the improvement of newborn care and outcomes.

## METHODS

### Search strategy and eligibility criteria

This review was registered with PROSPERO (CRD42021248145) and reported according to the PRISMA 2020 Statement [23] (Tables S1, S2). We searched five databases: PubMed, WHO Global Index Medicus, EMBASE, Web of Science, and the Cochrane Library from inception to February 2021 with no language restrictions. The database searches were supplemented with hand-searching of reference lists of included studies and expert consultation. We applied search terms related to facility-level collection of data for neonatal indicators in LMIC (Table S3). This included terms related to neonates, infants or perinatal health, health facilities, routine data sources including registries, medical records, and aggregate reports, data quality or quality indicators, and all LMIC countries and related terms.

Inclusion criteria:

- conducted in LMIC setting, as defined by the World Bank [24];
- focusing on health facility setting of any type (public, private, not for profit, etc.) or level (primary, secondary, or tertiary hospitals, health centres, etc.);
- reporting quantitative data on availability and quality of data for newborn indicators (from birth to 28 days after delivery);
- observational study design (except case-control studies, case reports, or case series from individual patients) OR relevant baseline or control group data from interventional or quasi-experimental designs, and;
- reporting on data quality in:
  - individual patient case notes;
  - routine facility registers, or;
  - aggregate reports, including DHIS or HMIS reports.

Exclusion criteria:

- reporting only as abstracts or poster presentations;
- objective did not include assessment of quality of data for newborn indicators;
- results were aggregated with data from other age groups or from origins other than primary sources, or;
- data quality was assessed in non-routine data sources, including ad-hoc, project-specific registries or data sources not held at the hospital (i.e., patient-held child medical records).

### Data collection

Two authors (RL and IM) independently screened titles and abstracts of all identified records for eligibility using the online Abstrackr [25] tool, resolving any discrepancies in discussion with a third author (ML). Both authors independently reviewed the full-text articles for all relevant abstracts to determine eligibility. Up to three attempts were made by the researchers to contact authors of articles when additional information or clarification was needed to assess the inclusion of a publication.

Any discrepancies were resolved via discussion between the two researchers (RL and IM), with consensus sought from a third researcher (ML).

Two authors (RL and IM) independently extracted data from included articles using customized data abstraction forms in the Systematic Review Data Repository (SDRD) online platform [26]. Any discrepancies were resolved through discussion, with the involvement of a third author as necessary.

Information was extracted on study design, setting (country, region, number of health facilities by type/level, name of ward), populations whose primary data were assessed (eg, all births, all neonates admitted, etc.), and data sources which included: a) individual patient case notes, b) routine facility registers, or c) aggregate reports).

All available quantitative data on three dimensions of data quality were extracted. Definitions of these dimensions were adapted from the WHO Data Quality Review (DQR) [27] tool to encompass both aggregate reporting, as focused on in the WHO DQR, and individual-level data sources:

1. completeness of indicator data, defined as whether data for newborn indicators were recorded in individual or facility-level data sources, and timeliness of facility reporting, defined as whether data elements were reported in aggregate form within predefined deadlines;
2. internal consistency of indicator data, defined as coherence between related data elements captured in the same data source: including proportion of outliers, consistency between birth outcome or gestational age captured multiple times for the same patient, and birthweight heaping;
3. external consistency, defined as the level of agreement between two different data sources measuring the same newborn data element (for example, whether birth outcome obtained by direct observation agrees with what is recorded in primary facility source).

Available quantitative data on other measures of data quality (eg, presence of registers or records, observed births recorded, data illegibility, partograms completed according to standard protocol, incorrectly coded data, data meeting specified quality standards, aggregate reports submitted on time) were also extracted from included articles.

Definitions of reported quality measures and tools or methods used to evaluate them were also collected, along with any measures of variance (standard deviations, 95% confidence intervals, etc.). Data were extracted as reported in the results section of each article and subsequently converted as needed (eg, percent of incomplete data recorded converted to percent of complete data). Authors of six articles [28-33] were contacted for additional information, among whom one [28] responded.

### Risk of bias assessment

As only observational studies or baseline or control group data from interventional and quasi-experimental studies are included in the current review, several tools outlined in a recent review of methodological quality and risk of bias assessment tools for primary and secondary medical studies were considered to evaluate risk of bias or quality of evidence [34].

The Critical Appraisal Tool to assess the Quality of Cross-Sectional Studies (AXIS) [35] was chosen as suitably adaptable to the current review because 19 of 20 evaluation criteria were relevant to included studies. These criteria include assessment of study aims, objectives and design, sample size justification, sample representativeness of clearly defined target population, sample selection process, attention to and information on missing data, correlation of measures and aims and of results and methods, clear methods, statistical analysis and definition of statistical significance, data description, internal consistency, conclusion justified by results, and inclusion of limitations, conflicts of interest, and ethics approval information. The following minor adaptations were made to the AXIS tool, as appropriate to our research question:

- score modified from 20-point system to 19-point system, removing item on non-response bias as this is not relevant to included studies reviewing routine health facility data sources;
- reference to “non-responders” in two items was changed to “non-recorded data elements or patient records”, as the current review focused on routine health facility data rather than surveys;
- removal of reference to “risk factor” from two items, as no risk factors were evaluated in this review.

Two authors (RL and IM) independently assessed risk of bias using the adapted AXIS tool, with discordance resolved via discussion [RL, IM, and KP]. Adapted quality categories were applied based on ranges in another review using the 20-point AXIS tool [36]. Scores >14 were considered high quality, from 9-14 moderate quality, and <9 low quality.

### Statistical analyses

Heterogeneity of results for each quality measure reported in more than one study was assessed using the I^2^ value, with values between 25%-50% considered low, between 50% and 75% intermediate, and >75% high [37].

Meta-analysis was not performed because heterogeneity was >75% for all quality measures [38]. As such, descriptive analysis was conducted, with data synthesized and visualized using tables and figures. Results were first grouped by data quality dimension – 1) completeness and timeliness, 2) internal consistency, 3) external consistency, and 4) other measures of data quality. Within each group, results were subsequently organized by data source level – 1) individual level, including both individual case notes and routine facility registers, and 2) facility level, including aggregate reports. Within each of these groups, the most frequently reported specific data quality measures were summarized graphically, with additional measures described in tables and text.

All analyses were conducted in R (R Core Team, Vienna, Austria, 2017) using the ggplot2, ggalt and ggfort packages for figure development.

## RESULTS

**Figure 1:**
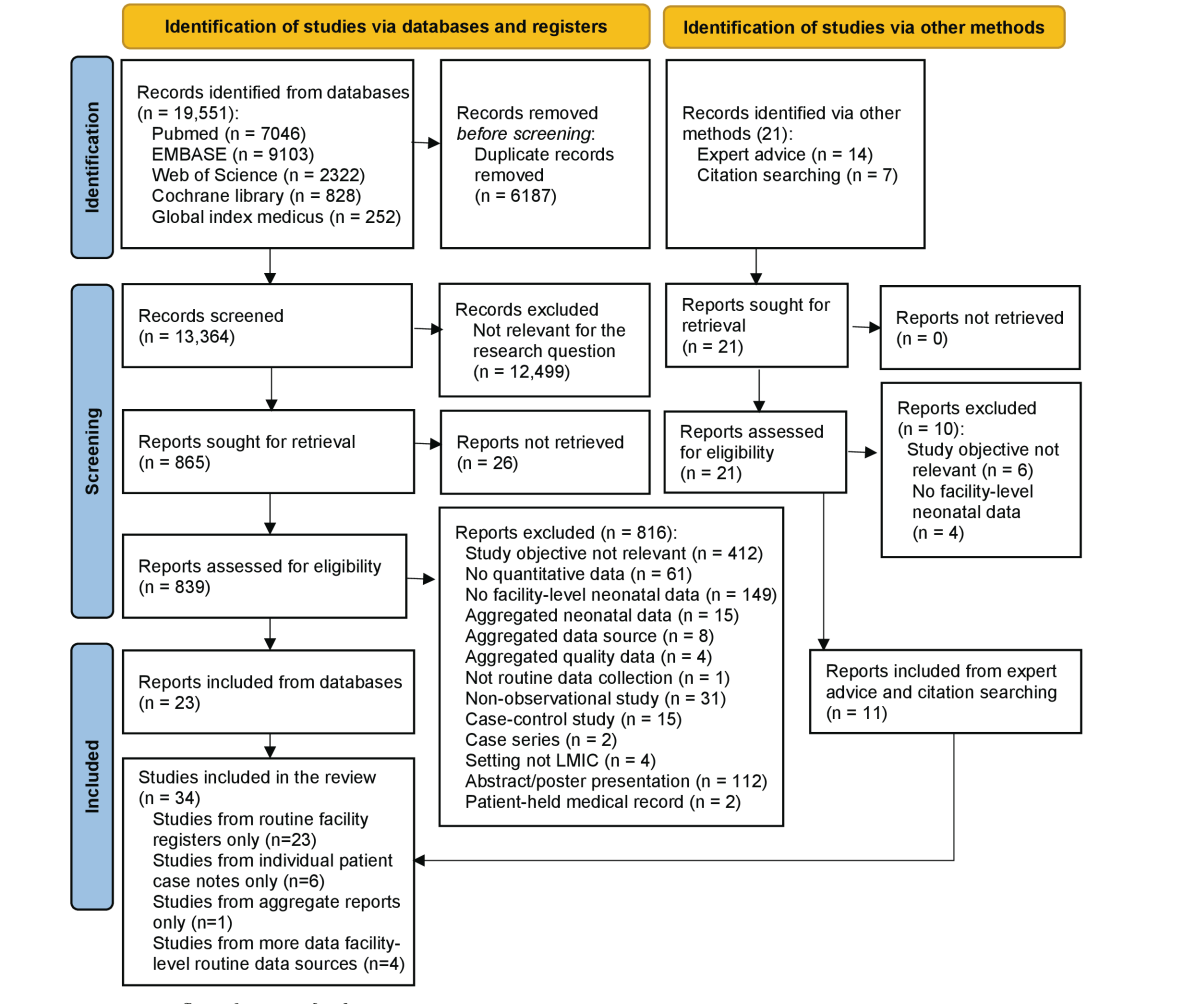
PRISMA flow diagram [23].

Among the 19 572 articles identified, 34 studies [28,30-33,39-67] were included in the systematic review (**Figure 1**, Table S4 in the **Online Supplementary Document**). 23 included studies were identified through database review and 11 came from expert recommendations solicited by the study team.

### Characteristics of included studies

**Table 1** summarizes the main characteristics of included studies, with detailed information available in Table S4 in the **Online Supplementary Document**.

**Table 1:** Main characteristics of included studies (n = 34).

Characteristic of included studies	Number of articles (%)
**Publication year:**	
2006-2010	5 (14.7%)
2011-2015	8 (23.5%)
2016-2020	15 (44.1%)
2021	6 (17.6%)
**Study design:**	
Cross-sectional	28 (82.4%)
Quasi-experimental	4 (11.8%))
Cohort	1 (2.9%)
Nested observational	1(2.9%)
WHO geographical region:*	
Africa	30 (88.2%)
South-East Asia	24 (70.6%)
Eastern Mediterranean	2 (5.9%)
Western Pacific	1 (2.9%)
Americas	0 (0.0%)
Europe	0 (0.0%)
**Type of health facility:**	
Hospital	26 (76.5%)
Clinic	5 (14.7%)
Health center	6 (17.6%)
**Health facility financing:**	
Public	27 (79.4%)
Faith-based	1 (2.9%)
Public-private mix	2 (5.9%)
Public-faith-based mix	2 (5.9%)
Public-private-faith-based mix	2 (5.9%)
**Level of health facility:†**	
Primary	5 (14.7%)
Secondary	3 (8.8%)
Tertiary	3 (8.8%)
District	5 (14.7%)
Referral	4 (11.8%)
Training/University	2 (5.9%)

*More than one region can be represented in multi-country studies.

†Facility level not always recorded in standardized categories.

**Figure 2:**
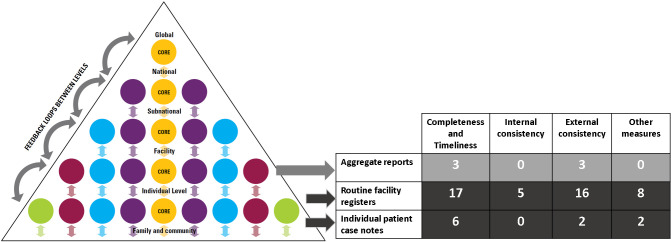
Data sources, adapted WHO DQR data quality dimensions, and number of studies reporting each dimension (n = 34).

Most studies assessed individual-level data (lowest level of data source pyramid, **Figure 2**), usually from routine facility registers (n = 23), with only six studies assessing individual patient case notes, two reporting on data in both registers and case notes, two on data from case notes and aggregate reports, and one on aggregate report data quality (**Figure 2**, Table S4 in the **Online Supplementary Document**).

Included studies were found to have only assessed data used to calculate neonatal indicators rather than the indicators themselves, which require both a numerator and denominator. The most commonly included data element for newborn indicators was birth outcome (7 studies), followed by birthweight (6 studies), with 16 data elements assessed in only one study (Tables S5-S8 in the **Online Supplementary Document**).

Data quality measures reportedly used in included studies varied. Measures of data completeness utilized in these articles included percent completeness for individual data elements and groupings of selected data elements, percent of reports completed correctly, and average percent of data element completeness across multiple facilities. Timeliness of reporting of aggregate data from primary facility sources was assessed in one study. Measures of internal consistency used in these studies included birthweight heaping, inconsistencies between two data elements in the same record or register entry, and outliers. Sensitivity, specificity, and percent agreement were most commonly used by authors of included studies to assess external consistency, with additional measures including area under the receiver operator curve (AUC), inflation factor, validity ratio, correlation, absolute difference, positive and negative percent discordance, and inter-class correlation coefficient. Other measures of data quality reported in these studies were found, including presence of registers or records, observed births recorded, data illegibility, partograms completed according to standard protocol, incorrectly coded data, data meeting specified quality standards, and aggregate reports submitted (Tables S5-S8 in the **Online Supplementary Document**). We have synthesized and described the results from these varied measures in the following figures, tables, and text.

### Quality of methodology in included studies

A summary of quality evaluations of the methodology of included studies as assessed by the modified AXIS tool is shown in **Table 2**.

**Table 2:** Summary of quality of methodology of included studies using modified AXIS tool (n = 34)

Quality score*	Quality rating*	Number of studies (%)	References
19	High	1 (2.9%)	[32]
18	High	12 (35.3%)	[31,39-46,48-50]
17	High	10 (29.4%)	[28,52,54-61]
16	High	5 (14.7%)	[51,53,62-64]
15	High	4 (11.8%)	[30,47,65,66]
14	Moderate	2 (5.9%)	[33,67]

*Quality score of the modified AXIS tool ranges from 0 to 19 with scores >14 rated as high quality, from 9-14 moderate quality, and <9 low quality.

The most common quality issue was lack of information provided on missing data (8 studies), followed by missing justification for sample size calculation (6 studies), no information provided on conflicts of interest or existing conflicts of interest (5 studies), no discussion of limitations (3 studies), no information on ethics approval or informed consent (2 studies), and lack of adequate description of sampling (1 study) (Table s9 in the **Online Supplementary Document**).

### Data completeness and timeliness

Overall, 22 studies reported on data completeness or timeliness of data for newborn indicators (Table S5 in the **Online Supplementary Document**).

### Individual patient case notes and routine facility registers

**Figure 3:**
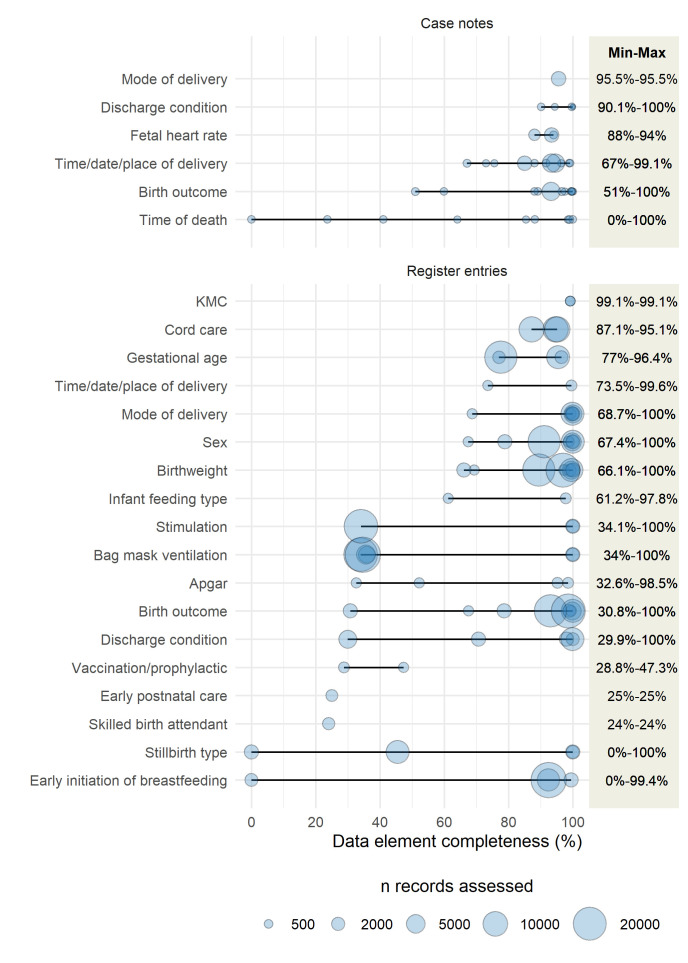
Completeness of individual neonatal data elements in case notes and facility registers across studies. KMC – Kangaroo Mother Care. Figure shows results from 17 studies, six from case notes [28,57,60,62,65,66] and eleven from registers [39,40,42-46,52,55,58,61].

**Figure 3** synthesizes results from 17 studies assessing the completeness of data for newborn indicators (six from case notes and nine from registers). Data not included in the figure are summarized in **Table 3** and subsequent text and in Table S5 in the **Online Supplementary Document**. The sample size of register entries or case notes assessed varied greatly across the 17 included studies, from 49 to 22 393. Included studies assessed 19 data elements overall (three in case notes only, 13 in register entries only, three in both) including early postnatal care, presence of a skilled birth attendant, infant feeding type, cord care, time of death, vaccination/prophylactic, and type of stillbirth in one study each, and birthweight in six studies (**Figure 3**, Table S5 in the **Online Supplementary Document**).

**Table 3:** Completeness of composite data elements or single data elements across multiple facilities in case notes and facility registers

Data element assessed	Range, % completeness	n studies (%)	References
Composite data elements	20.0%-96.9%	4 (11.8%)	[31,48,52,57]
APGAR score, averaged across centers	93.0%-99.0%	1 (2.9%)	[32]
Birthweight, averaged across centers	87.0%-97.0%	1 (2.9%)	[32]
Discharge status, averaged across centers	55.0%-91.0%	1 (2.9%)	[32]
Gestational age, averaged across centers	52.0%-92.0%	1 (2.9%)	[32]

Case notes assessments reported most frequently on completeness of time/date/place of delivery, birth outcome, and fetal heart rate (3 studies each), with only a single study reporting on each of the remaining three data elements. Percent completeness was reported to be greater than 80% for mode of delivery, discharge condition, and fetal heart rate, while wider ranges of percent completeness were reported for time/date/place of delivery (67%-91%), birth outcome (51%-100%), and time of death (0%-100%) (**Figure 3**, Table S5 in the **Online Supplementary Document**).

Routine register assessments reported most frequently on completeness of birthweight (5 studies), followed by gestational age, sex, and bag-mask ventilation (3 studies each), with one to two studies reporting on the remaining 12 data elements. Reported percent completeness was always greater than 60% for cord care, gestational age, time/date/place of delivery, sex, birth weight, and infant feeding type and less than 50% for vaccination or prophylaxis, early postnatal care, or presence of a skilled birth attendant. All other data elements exhibited wider reported ranges of completeness, ranging from 0%-100% by study for stillbirth type and 34.1%-100% by study for stimulation (**Figure 3**, Table S5 in the **Online Supplementary Document**).

Data from five studies reporting on completeness of data for newborn indicators found in register entries and case notes not included in **Figure 3** because they reported on composite data elements combining two or more individual data elements or average completeness across multiple facilities are summarized in **Table 3** (Table S5 in the **Online Supplementary Document**).

### Aggregate reports

Two studies not represented in **Figure 3** reported on completeness of aggregate reports: 68.4% completeness of submission date in reports from the MCH unit to the district office was observed in one [53], and completeness of reporting of newborn data elements in DHIS2 using aggregate data from primary facility sources ranged from 20% for exclusive breastfeeding to 54% for polio vaccination in another [64] (Table S5 in the **Online Supplementary Document**).

One of these studies also assessed the timing of regular reports of aggregated data from primary sources, finding 84% were submitted on time [64] (Table S5 in the **Online Supplementary Document**).

### Internal consistency

**Figure 4:**
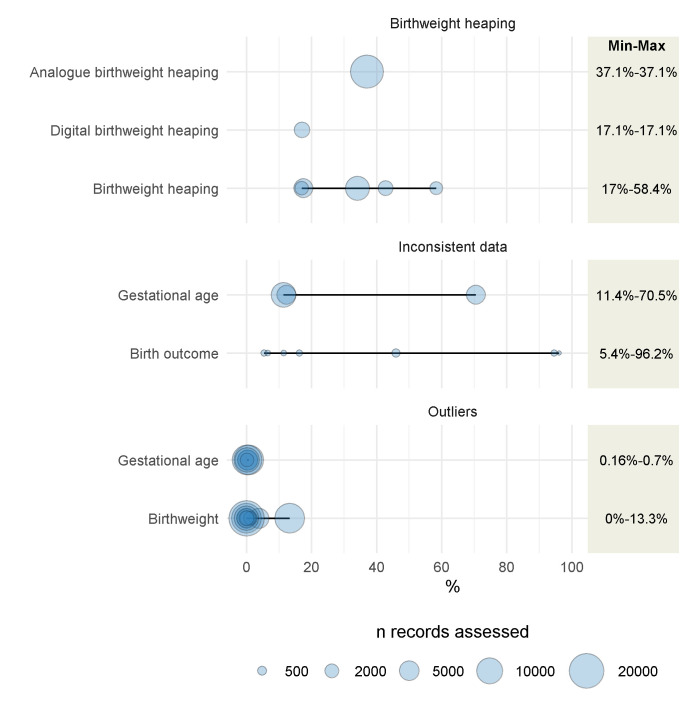
Internal consistency of newborn data elements recorded in routine facility registers across studies. Figure shows results from four studies [43,44,47,58].

**Figure 4** synthesizes data from four studies reporting on internal consistency, all focusing on routine registers and birthweight or gestational age. Data from one study that was not included are summarized after **Figure 4** and in Table S6. The sample sizes in these studies ranged from 26 to 17 631 entries assessed. Data consistency (5.4% to 96.2%) and birthweight heaping (17.1% to 58.43%) were highly heterogenous, while frequency of outliers ranged from 0.0% to 13.3%. The range of internal consistency estimates varied from 0.2%-0.8% for gestational age outliers to 5.4%-96.2% for inconsistent birth outcome data (**Figure 4**, Table S6 in the **Online Supplementary Document**).

One study not included in **Figure 4** reported on median difference between recorded gestational age and gestational age calculated using the date of last menstrual period (1.7 weeks [IQR 3.9]) [58] (Table S6 in the **Online Supplementary Document**).

### External consistency

#### Individual patient case notes and routine facility registers

Overall, a total of 18 studies reported on external consistency of data for newborn indicators in individual patient case notes and routine facility registers, 11 comparing facility register data with direct observation, seven comparing facility register data with death audits, MCH and HMIS reports, or capture-recapture estimates, one comparing individual patient case notes with direct observation, and one comparing maternal recall to district health centre reports. Included articles assessed 16 data elements for external consistency, with study authors employing twelve different measures. Birth outcome was the data element for which external consistency was most often reported (5 studies), followed by neonatal death and early breastfeeding initiation (3 studies each), skilled birth attendant, bag-mask ventilation, birth weight, cord care, Kangaroo Mother Care (KMC) initiation, mode of delivery, early postnatal care, and nevirapine prophylaxis (2 studies each), and asphyxia, stimulation, dry and wrap newborn, gestational age, and essential newborn care (1 study each). Specificity, sensitivity, and percent agreement were the measures of external consistency most frequently reported in included studies (9, 11, and 12 studies, respectively) (Table S7 in the **Online Supplementary Document**).

**Figure 5:**
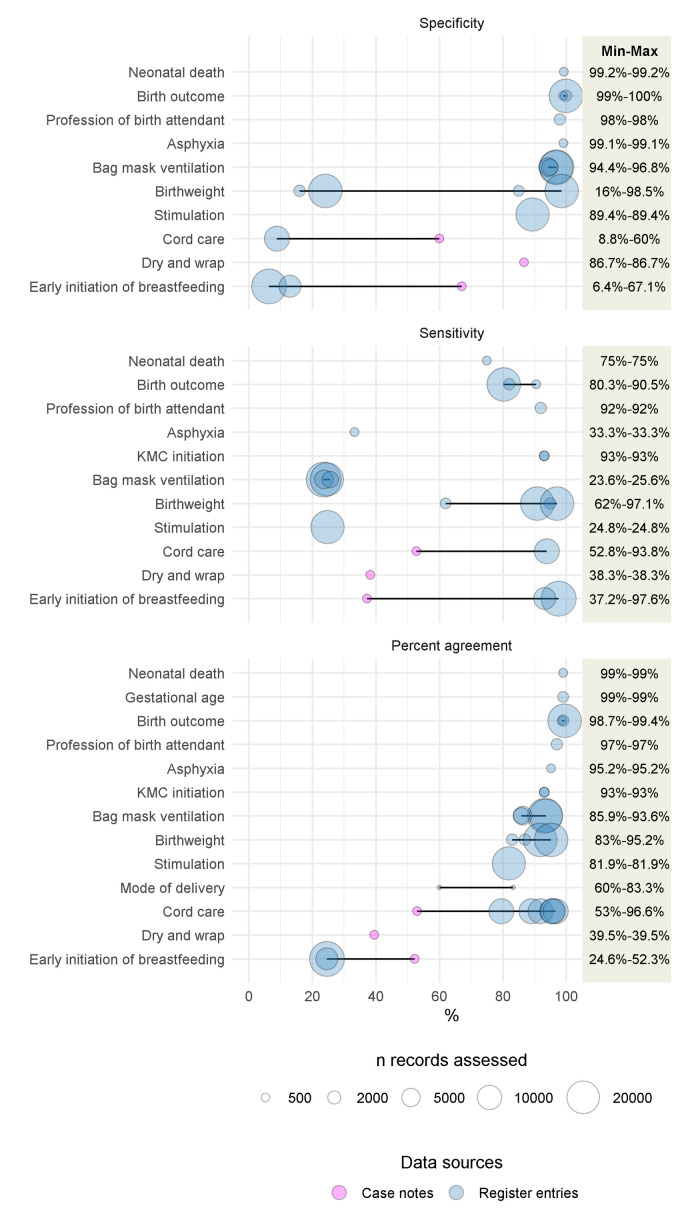
External consistency of neonatal data elements in facility registers with direct observation across studies. KMC – Kangaroo Mother Care. Figure show results from 10 studies, 9 reporting on specificity (1 study from case notes [54] and 8 from registers [39,41-43,45,46,52,54]), 10 reporting on sensibility (1 study from case notes [54] and 9 from registers [39-43,45,46,52,54]), 10 reporting on percent agreement (1 study from case notes [54] and 9 from registers [39,41-43,45,46,52,54,67]).

**Figure 5** summarizes findings from the 10 studies reporting on specificity, sensitivity, and/or percent agreement of newborn data elements in facility registers (n = 10) or case notes (n = 1) compared with direct observation. Study size varied from 57 to 22 393 document entries assessed in these 10 studies, and birth outcome was most frequently assessed (4 studies), followed by early breastfeeding initiation (3 studies), bag-mask ventilation, birth weight, cord care, KMC initiation, (2 studies each), asphyxia, profession of birth attendant, mode of delivery, gestational age, stimulation, dry and wrap neonate, and neonatal death (1 study each). There was high heterogeneity in reported specificity (6.0% to 100%), sensitivity (23.6% to 97.6%), and percent agreement (24.6% to 99.4%). Considering individual data elements, birth outcome specificity had the narrowest range (98.8%-100.0%) and essential newborn care specificity the widest (6.0%-86.8%) (**Figure 5**, Table S7 in the **Online Supplementary Document**). Data not included in **Figure 5** are summarized in **Table 4** and subsequent text and presented in detail in Table S7 in the **Online Supplementary Document**.

**Table 4:** Other measures of external consistency of neonatal data elements in individual case notes or facility registers with direct observation.

External consistency measure	External consistency (range)	n studies (%)	References
Validity ratio	0.85-4.29	3 (8.8%)	[42,43,45]
AUC	0.52-0.95	2 (5.9%)	[52,54]
Inflation factor	0.97-3.22	1 (2.9%)	[52]
Positive discordance	0.6%-82.8%	1 (2.9%)	[54]

Additional data on external consistency of registry entries or case notes with direct observation from five studies were excluded from **Figure 5** because they reported on other measures of external consistency (**Table 4**) or composite data elements (Table S7 in the **Online Supplementary Document**).

The composite essential newborn care data element combining immediate breastfeeding initiation and keeping the baby warm was reported to have 6% specificity, 97% sensitivity, and 44% agreement [52] (Table S7 in the **Online Supplementary Document**).

### Aggregate reports

Eight studies compared individual-level data sources with aggregate data and were not included in **Figure 5** (**Table 5**, Table S7 in the **Online Supplementary Document**).

**Table 5:** External consistency of individual level neonatal data elements with aggregate data.

Data source	Comparison data source	External consistency measures	External consistency (range)	n studies (%)	References
Register entries	Death audit	Specificity	97.7%-100.0%	1 (2.9%)	[50]
Register entries	Death audit	Sensitivity	97.5%-100.0%	1 (2.9%)	[50]
Register entries	Facility reports*	% agreement	6.25%-6.25%	1 (2.9%)	[61]
Register entries	Facility reports*	ICC	0.21-0.48	1 (2.9%)	[51]
Register entries	Facility reports*	Correlation	1.00-1.00	1 (2.9%)	[33]
Register entries	Facility reports*	Average deviation	51.7%-51.7%	1 (2.9%)	[30]
Register entries	Facility reports*	% within 10% average deviation	17.5%-17.5%	1 (2.9%)	[30]
Register entries	Capture-recapture	Sensitivity	52.0%-87.0%	1 (2.9%)	[63]
Facility records	Government administrative data	Difference	59.52-459.23	1 (2.9%)	[59]
Maternal recall	Facility reports*	% agreement	20.0%-20.0%	1 (2.9%)	[64]

*Facility reports included Maternal and Child Health reports, HMIS reports, District Health Center reports, DHIS2 monthly reports, and monthly reports.

## Other data quality measures

### Individual patient case notes and routine facility registers

Thirteen studies summarized in **Table 6** reported on data quality measures that did not fall within the three dimensions assessed in our review applied to data in individual patient case notes and routine facility registers (Table S8 in the **Online Supplementary Document**).

**Table 6:** Other measures of quality of neonatal data elements in facility registers and case notes

Data quality measure	Data quality estimate (range)	n studies (%)	References
% hospitals with registers	0.00%-100.0%	1 (2.9%)	[49]
% observed births recorded	80.7%-80.7%	1 (2.9%)	[67]
% maternal files found for c-section deliveries	100.0%-100.0%	1 (2.9%)	[57]
% illegible data	0.03-0.40	8 (23.5%)	[39-43,45,46,55]
% partograms not completed according to standards	86.0%-86.0%	1 (2.9%)	[66]
% incorrectly coded data	0.1%-3.7%	1 (2.9%)	[55]
% high quality records	6.7%-6.7%	1 (2.9%)	[56]

### Aggregate reports

Two studies assessed the delivery of regular reports of aggregated data from primary facility sources, finding 75%-84% of reports existed [53,64] (Table S8 in the **Online Supplementary Document**).

## DISCUSSION

We found 34 studies in the published literature that have evaluated the quality of newborn data elements routinely collected at facility level in LMIC, and no study reporting on the quality of newborn indicators. This systematic review highlights heterogeneity in both data quality and methods used to assess data quality.

The studies included in this systematic review were not fully representative of all regions or health system levels where newborn babies receive care in LMICs. Identified studies were all relatively recent, from 2007 onwards, although there were no date limitations in the search strategy. The greatest frequency of publications on this topic occurred in 2020 and 2021, many resulting from the Every Newborn-BIRTH Indicators Research Tracking (EN-BIRTH) study (n = 9). This observational study in five hospitals in Bangladesh, Nepal, and Tanzania compared hospital register and exit survey data to gold standard direct observation or case note verification data for maternal and newborn indicators [45]. Notably, no published peer-reviewed studies reported on newborn data quality in WHO Americas or European regions. Most studies were conducted in public health facilities (79%). The small number of studies identified in the published literature does not seem to reflect the investments made to improve facility-level data collection in LMIC, including the DHIS and DHIS2 systems. Opportunities exist to strengthen the peer-reviewed literature in this area.

The quality of methodology as evaluated by a modified version of the AXIS tool was high for all but one article included in this review, which was rated of moderate quality. These high to moderate assessments are reassuring, though other more relevant assessment tools might enable greater distinction between articles of the type included in this review. The AXIS tool was more applicable to the current review than other tools developed to assess the methodology of observational studies [68-76], which focused on items irrelevant to this review such as outcome or exposure measures and definition of comparison groups. At the same time, the AXIS tool did not include consideration of factors that might be important to capture related to the methodology of data quality assessment studies, such as whether standardized methodology was used.

The data sources evaluated in included studies were predominantly registers, with very few assessing individual patient case notes directly. Several studies also assessed aggregate reports such as those included in DHIS [30,33,50,51,53,61,63,64,59]. Only a few studies explored whether assessment of quality of specific data for newborn indicators was feasible given the design of registers or case notes [39,41,42,44-46]. For instance, in register designs where instructions are to leave blank if not done, it is impossible to discern whether a blank is truly not done or is incomplete, which can impact data quality [45]. These gaps indicate an opportunity to expand research on different types of individual-level routine health facility data sources and investigation of factors influencing data quality, reporting and use of data at all levels.

Data elements assessed for quality in routine documents varied, with birthweight [32,43,44,52,55,58] or gestational age [44,52,58] reported most frequently, while many other key neonatal data elements were only reported from a single study, including KMC initiation [40], neonatal death [54], early postnatal care [61], and presence of a skilled birth attendant [61]. Our systematic review identified that the quality of other key data elements needed for core newborn indicator measurement, including antenatal corticosteroid use and treatment of severe neonatal infections, have not yet been assessed in the identified published literature. Given the importance of routine measurement to track progress towards improved neonatal outcomes, particularly in LMICs, more research is needed on the quality of data routinely collected in facility settings for all newborn core indicators, as well as factors influencing data quality, and strategies to address barriers to the collection of high-quality data. Future studies may focus both on regions and countries where few studies have been conducted, and on countries already committed to improving data, where there are concrete opportunities for improvement.

Methods and measures used to assess the quality of newborn data elements varied widely across identified studies. The numbers of centres and individual patient entries assessed were heterogeneous, and eligibility criteria ranged from very narrowly defined populations, such as women undergoing planned cesarean section, to all women delivering or all babies delivered at participating facilities. Several included studies assessed the quality of composite data elements, which did not permit the identification of specific data elements with quality issues. Though the Performance of Routine Information System Management (PRISM) Tools [78] were mentioned in some articles, none made use of this or other tools like the WHO DQR [27], which were specifically developed to guide evaluations of routine health information system data quality. Results of assessments using these resources can be found in the grey literature [79,80]. In the case of the WHO DQR, the fact that recommended core indicators do not include neonatal indicators may impact the use of this tool to assess routine facility data in this specific field. Opportunities exist to further leverage the PRISM framework and WHO DQR, expanded beyond recommended core indicators, to evaluate routine health facility data for newborn indicators in LMIC and publish results in the peer-reviewed literature. The use of more standardized methods for assessing data quality could in turn improve interpretation and comparison of results across studies and settings, greatly increasing its usefulness to inform interventions and investments.

While measures of completeness were common (21/34 included studies) and the same across studies, only one study assessed timeliness of aggregate reports. Only four studies reported on internal consistency using varied measures, with three studies each reporting on outliers and inconsistent data and two studies on birthweight heaping. More studies assessed external consistency (n = 18), but these demonstrated notable heterogeneities in measures used. While ten studies reported sensitivity, specificity, or percent agreement, nine other measures of external consistency were also used (validity ratio, area under the receiver operator curve (AUC), inflation factor, correlation, interclass correlation coefficient (ICC), positive discordance, difference, average deviation, and percent with greater than 10% average deviation). Comparisons were made between register entries or case notes and five different data sources - direct observation, death audit, government administrative data, aggregate reports (including DHIS2 and HMIS data), and capture-recapture estimates. The most commonly reported measures – sensitivity, specificity, and percent agreement – indicated very different results for the same data element across different facilities and different studies. Four other measures of data quality were assessed: illegibility in seven studies, incorrectly coded data in two, and register availability and data quality according to pre-established criteria in one study each.

To our knowledge, no systematic review has assessed the quality of health facility data in general in LMIC. A peer-reviewed publication of an assessment carried out in 115 health facilities in Tanzania reported varied agreement between different data sources across areas of care and different indicators, with lowest agreement between individual-level data sources like registers and tally sheets, and between these sources and facility-level reports in DHIS2 [81]. These findings are echoed by a case series in the literature comparing data on four common under-five children’s illnesses in outpatient registers and monthly reports in Tanzania that found low completeness and timeliness of reporting and over-reporting of diagnoses [82]. Similarly, an HMIS review in Ethiopia using PRISM tools found lower register completeness compared to facility report completeness, and low data accuracy comparing reports to registers [83]. One systematic review focused specifically on childhood vaccination data quality in LMIC, comparing health facility data with patient recall, home-based records, and serology, as well as different combinations of data sources with one another, finding that facility data generally had better agreement with serology than surveys or home-based records [84]. While results from the review of vaccination data from different sources appear to reiterate our finding that high-quality facility-level data can be collected in LMIC settings, the individual studies in Tanzania and Ethiopia highlight the variability and potential issues with data quality both in individual-level facility data and aggregate reports of these data. Action is needed to ensure not only the quality of facility-level data but also of reporting up through the levels of the health system.

While this review employed standardized PRISMA methods, only peer-reviewed publications were included, and authors acknowledge that the omission of unpublished grey literature was a limitation. Non-published data on newborn indicators collected at the facility level has been covered in part by existing reviews [85]. Grey literature results are in line with findings from the current review, with poor availability of key newborn indicators at the national level and poor or very poor data quality reported as a factor affecting HMIS data use and quality in 18/23 countries [85]. This review was also limited by the heterogeneity of reported data, which did not allow for meta-analysis.

## CONCLUSIONS

In conclusion, this systematic review shows opportunities to expand and further standardize the published peer-reviewed literature on the quality of routine facility data for newborn indicators in LMIC. Robust evidence is needed to drive policy around data quality initiatives and ultimately contribute to reductions in newborn morbidity and mortality in these high burden settings.

## Additional material


Online Supplementary Document
