# Supplementary material for: Quality of routine health facility data used for newborn indicators in low- and middle-income countries: A systematic review
Source: J Glob Health. 2022 Apr 23;12:04019. doi: 10.7189/jogh.12.04019 (PMC9031513; doi:10.7189/jogh.12.04019)
Supplement: Online Supplementary Document [file jogh-12-04019-s001.pdf]

Table S1: PRISMA 2020 Checklist

Section and Topic	Item #	Checklist item	
TITLE			
Title	1	Identify the report as a systematic review.	X
ABSTRACT			
Abstract	2	See the PRISMA 2020 for Abstracts checklist.	X
INTRODUCTION			
Rationale	3	Describe the rationale for the review in the context of existing knowledge.	X
Objectives	4	Provide an explicit statement of the objective(s) or question(s) the review addresses.	X
METHODS			
Eligibility criteria	5	Specify the inclusion and exclusion criteria for the review and how studies were grouped for the syntheses.	X
Information sources	6	Specify all databases, registers, websites, organisations, reference lists and other sources searched or consulted to identify studies. Specify the date when each source was last searched or consulted.	X
Search strategy	7	Present the full search strategies for all databases, registers and websites, including any filters and limits used.	X
Selection process	8	Specify the methods used to decide whether a study met the inclusion criteria of the review, including how many reviewers screened each record and each report retrieved, whether they worked independently, and if applicable, details of automation tools used in the process.	X
Data collection process	9	Specify the methods used to collect data from reports, including how many reviewers collected data from each report, whether they worked independently, any processes for obtaining or confirming data from study investigators, and if applicable, details of automation tools used in the process.	X
Data items	10a	List and define all outcomes for which data were sought. Specify whether all results that were compatible with each outcome domain in each study were sought (e.g. for all measures, time points, analyses), and if not, the methods used to decide which results to collect.	X

	10b	List and define all other variables for which data were sought (e.g. participant and intervention characteristics, funding sources). Describe any assumptions made about any missing or unclear information.	X
Study risk of bias assessment	11	Specify the methods used to assess risk of bias in the included studies, including details of the tool(s) used, how many reviewers assessed each study and whether they worked independently, and if applicable, details of automation tools used in the process.	X
Effect measures	12	Specify for each outcome the effect measure(s) (e.g. risk ratio, mean difference) used in the synthesis or presentation of results.	X
Synthesis methods	13a	Describe the processes used to decide which studies were eligible for each synthesis (e.g. tabulating the study intervention characteristics and comparing against the planned groups for each synthesis (item #5)).	X
	13b	Describe any methods required to prepare the data for presentation or synthesis, such as handling of missing summary statistics, or data conversions.	X
	13c	Describe any methods used to tabulate or visually display results of individual studies and syntheses.	X
	13d	Describe any methods used to synthesize results and provide a rationale for the choice(s). If meta-analysis was performed, describe the model(s), method(s) to identify the presence and extent of statistical heterogeneity, and software package(s) used.	X
	13e	Describe any methods used to explore possible causes of heterogeneity among study results (e.g. subgroup analysis, meta-regression).	X
	13f	Describe any sensitivity analyses conducted to assess robustness of the synthesized results.	
Reporting bias assessment	14	Describe any methods used to assess risk of bias due to missing results in a synthesis (arising from reporting biases).	X
Certainty assessment	15	Describe any methods used to assess certainty (or confidence) in the body of evidence for an outcome.	X
RESULTS			

Study selection	16a	Describe the results of the search and selection process, from the number of records identified in the search to the number of studies included in the review, ideally using a flow diagram.	X
	16b	Cite studies that might appear to meet the inclusion criteria, but which were excluded, and explain why they were excluded.	X
Study characteristics	17	Cite each included study and present its characteristics.	X
Risk of bias in studies	18	Present assessments of risk of bias for each included study.	X
Results of individual studies	19	For all outcomes, present, for each study: (a) summary statistics for each group (where appropriate) and (b) an effect estimate and its precision (e.g. confidence/credible interval), ideally using structured tables or plots.	X
Results of syntheses	20a	For each synthesis, briefly summarise the characteristics and risk of bias among contributing studies.	X
	20b	Present results of all statistical syntheses conducted. If meta-analysis was done, present for each the summary estimate and its precision (e.g. confidence/credible interval) and measures of statistical heterogeneity. If comparing groups, describe the direction of the effect.	X
	20c	Present results of all investigations of possible causes of heterogeneity among study results.	X
	20d	Present results of all sensitivity analyses conducted to assess the robustness of the synthesized results.	X
Reporting biases	21	Present assessments of risk of bias due to missing results (arising from reporting biases) for each synthesis assessed.	X
Certainty of evidence	22	Present assessments of certainty (or confidence) in the body of evidence for each outcome assessed.	X
DISCUSSION			
Discussion	23a	Provide a general interpretation of the results in the context of other evidence.	X
	23b	Discuss any limitations of the evidence included in the review.	X
	23c	Discuss any limitations of the review processes used.	X

	23d	Discuss implications of the results for practice, policy, and future research.	X
OTHER INFORMATION			
Registration and protocol	24a	Provide registration information for the review, including register name and registration number, or state that the review was not registered.	X
	24b	Indicate where the review protocol can be accessed, or state that a protocol was not prepared.	X
	24c	Describe and explain any amendments to information provided at registration or in the protocol.	X
Support	25	Describe sources of financial or non-financial support for the review, and the role of the funders or sponsors in the review.	X
Competing interests	26	Declare any competing interests of review authors.	X
Availability of data, code and other materials	27	Report which of the following are publicly available and where they can be found: template data collection forms; data extracted from included studies; data used for all analyses; analytic code; any other materials used in the review.	X

From: Page MJ, McKenzie JE, Bossuyt PM, Boutron I, Hoffmann TC, Mulrow CD, et al. The PRISMA 2020 statement: an updated guideline for reporting systematic reviews. [illegible] 10.1136/bmj.n71

For more information, visit: http://www.prisma-statement.org/

Table S2: PRISMA 2020 for Abstracts Checklist

Section and Topic	Item #	Checklist item	
TITLE			
Title	1	Identify the report as a systematic review.	X
BACKGROUND			
Objectives	2	Provide an explicit statement of the main objective(s) or question(s) the review addresses.	X

METHODS			
Eligibility criteria	3	Specify the inclusion and exclusion criteria for the review.	X
Information sources	4	Specify the information sources (e.g. databases, registers) used to identify studies and the date when each was last searched.	X
Risk of bias	5	Specify the methods used to assess risk of bias in the included studies.	X
Synthesis of results	6	Specify the methods used to present and synthesise results.	X
RESULTS			
Included studies	7	Give the total number of included studies and participants and summarise relevant characteristics of studies.	X
Synthesis of results	8	Present results for main outcomes, preferably indicating the number of included studies and participants for each. If meta-analysis was done, report the summary estimate and confidence/credible interval. If comparing groups, indicate the direction of the effect (i.e. which group is favoured).	X
DISCUSSION			
Limitations of evidence	9	Provide a brief summary of the limitations of the evidence included in the review (e.g. study risk of bias, inconsistency and imprecision).	X
Interpretation	10	Provide a general interpretation of the results and important implications.	X
OTHER			
Funding	11	Specify the primary source of funding for the review.	X
Registration	12	Provide the register name and registration number.	X

From: Page MJ, McKenzie JE, Bossuyt PM, Boutron I, Hoffmann TC, Mulrow CD, et al. The PRISMA 2020 statement: an updated guideline for reporting systematic reviews. [illegible]
10.1136/bmj.n71

For more information, visit: http://www.prisma-statement.org/

Table S3: Search strings

Database	Search terms	Results

EMBASE	('maternal child health care'/exp OR 'maternal child health care' OR 'newborn'/exp OR 'baby'/exp OR newborn OR newborns OR neonate OR neonates OR infant OR infants OR baby OR babies)<br>AND<br>('africa'/exp OR 'africa' OR 'african'/exp OR 'african' OR 'developing country'/exp OR 'developing country' OR 'developing countries'/exp OR 'developing countries' OR 'low income country'/exp OR 'low income country' OR 'low income countries' OR 'low-income setting' OR 'low-resource setting' OR 'middle income country'/exp OR 'middle income country' OR 'middle income countries' OR 'middle-income setting' OR 'middle-resource setting' OR 'low and middle income countries'/exp OR 'low and middle income countries' OR 'lmic' OR 'afghanistan'/exp OR 'afghanistan' OR 'guinea-bissau'/exp OR 'guinea-bissau' OR 'sierra leone'/exp OR 'sierra leone' OR 'benin'/exp OR 'benin' OR 'haiti'/exp OR 'haiti' OR 'somalia'/exp OR 'somalia' OR 'burkina faso'/exp OR 'burkina faso' OR 'north korea'/exp OR 'north korea' OR 'democratic people`s republic of korea'/exp OR 'democratic people`s republic of korea' OR 'south sudan'/exp OR 'south sudan' OR 'burundi'/exp OR 'burundi' OR 'liberia'/exp OR 'liberia' OR 'syrian arab republic'/exp OR 'syrian arab republic' OR 'syria'/exp OR syria OR 'central african republic'/exp OR 'central african republic' OR 'madagascar'/exp OR 'madagascar' OR 'tajikistan'/exp OR 'tajikistan' OR 'chad'/exp OR 'chad' OR 'malawi'/exp OR 'malawi' OR 'tanzania'/exp OR 'tanzania' OR 'republic of congo'/exp OR 'republic of congo' OR 'democratic republic of the congo'/exp OR 'democratic republic of the congo' OR 'mali'/exp OR 'mali' OR 'togo'/exp OR 'togo' OR 'eritrea'/exp OR 'eritrea' OR 'mozambique'/exp OR 'mozambique' OR 'uganda'/exp OR 'uganda' OR 'ethiopia'/exp OR 'ethiopia' OR 'nepal'/exp OR 'nepal' OR 'yemen'/exp OR 'yemen' OR 'republic of yemen'/exp OR 'republic of yemen' OR 'gambia'/exp OR 'gambia' OR 'the gambia'/exp OR 'the gambia' OR 'niger'/exp OR 'niger' OR 'guinea'/exp OR 'guinea' OR 'rwanda'/exp OR 'rwanda' OR 'angola'/exp OR 'angola' OR 'india'/exp OR 'india' OR 'papua new guinea'/exp OR 'papua new guinea' OR 'bangladesh'/exp OR 'bangladesh' OR 'indonesia'/exp OR 'indonesia' OR 'philippines'/exp OR 'philippines' OR 'bhutan'/exp OR 'bhutan' OR 'kenya'/exp OR 'kenya' OR 'sao tome and principe'/exp OR 'sao tome and principe' OR 'bolivia'/exp OR 'bolivia' OR 'kiribati'/exp OR 'kiribati' OR 'senegal'/exp OR 'senegal' OR 'cabo verde'/exp OR 'cabo verde' OR 'kyrgyzstan'/exp OR 'kyrgyzstan' OR 'kyrgyz republic'/exp OR 'kyrgyz republic' OR 'solomon islands'/exp OR 'solomon islands' OR 'melanesia'/exp OR 'melanesia' OR 'cambodia'/exp OR 'cambodia' OR 'laos'/exp OR 'laos' OR 'lao people`s democratic republic'/exp OR 'lao people`s democratic republic' OR 'sudan'/exp OR 'sudan' OR 'cameroon'/exp OR 'cameroon' OR 'lesotho'/exp OR 'lesotho' OR 'timor-leste'/exp OR 'timor-leste' OR 'comoros'/exp OR 'comoros' OR 'mauritania'/exp OR 'mauritania' OR 'tunisia'/exp OR 'tunisia' OR 'democratic republic congo'/exp OR 'democratic republic congo' OR 'congo-brazzaville'/exp OR 'congo-brazzaville' OR 'congo'/exp OR 'congo' OR 'micronesia'/exp OR 'micronesia' OR 'ukraina'/exp OR 'ukraina' OR 'cote

d`ivoire'/exp OR 'cote d`ivoire' OR 'moldova'/exp OR 'moldova' OR 'uzbekistan'/exp OR 'uzbekistan' OR 'djibouti'/exp OR 'djibouti' OR 'mongolia'/exp OR 'mongolia' OR 'vanuatu'/exp OR 'vanuatu' OR 'egypt'/exp OR 'egypt' OR 'morocco'/exp OR 'morocco' OR 'vietnam'/exp OR 'vietnam' OR 'el salvador'/exp OR 'el salvador' OR 'myanmar'/exp OR 'myanmar' OR 'west bank and gaza' OR 'west bank' OR 'gaza' OR 'eswatini'/exp OR 'eswatini' OR 'swaziland'/exp OR 'swaziland' OR 'nicaragua'/exp OR 'nicaragua' OR 'zambia'/exp OR 'zambia' OR 'ghana'/exp OR 'ghana' OR 'nigeria'/exp OR 'nigeria' OR 'zimbabwe'/exp OR 'zimbabwe' OR 'honduras'/exp OR 'honduras' OR 'pakistan'/exp OR 'pakistan')

AND

('health care facility'/exp OR 'health care facility' OR 'health care facilities' OR 'health facility' OR 'health facilities' OR 'facility-level' OR 'facility' OR 'facilities' OR 'hospital'/exp OR 'hospital' OR 'hospitals' OR 'hospital-level' OR 'centre level' OR 'center level' OR 'centre' OR 'center' OR 'centres' OR 'centers' OR 'clinic' OR 'clinics' OR 'birthing centre' OR 'birthing center' OR 'birthing centres' OR 'birthing centers' OR 'nursery' OR 'nurseries' OR 'primary health care unit' OR 'primary health care units' OR 'ambulatory care facility' OR 'ambulatory care facilities')

AND

((('quality indicator' OR 'quality indicators' OR 'indicator' OR 'indicators') AND ('health care quality'/exp OR 'health care quality' OR 'health care delivery'/exp OR 'health care delivery' OR 'health care'/exp OR 'health care' OR 'healthcare' OR (delivery AND health AND care))) OR 'health status indicator'/exp OR 'health status indicator' OR 'health status indicators' OR 'health indicators' OR 'benchmarking'/exp OR 'benchmarking' OR 'health indicators' OR 'health metrics' OR 'measure' OR 'data' OR 'data sources' OR 'primary data sources' OR 'health information system' OR 'health information systems' OR 'medical information system'/exp OR 'medical information system' OR 'nursing information system'/exp OR 'nursing information system' OR 'hospital information system' OR 'HIS' OR 'routine health information' OR 'routine health system data' OR 'routine health information system' OR 'RHIS' OR 'health facility data' OR 'routine facility data' OR 'health management information system' OR 'HMIS' OR 'medical record'/exp OR 'medical record' OR 'Medical Records' OR 'patient medical records' OR 'patient records' OR 'medical records' OR 'administrative data' OR 'hospital registries' OR 'DHIS' OR 'DHIS2' OR 'Registries' OR 'registries' OR 'registry' OR 'registers' OR 'register'/exp OR 'register')

AND

('availability' OR 'data accuracy'/exp OR 'data accuracy' OR 'accuracy' OR 'quality' OR 'quality assessment' OR ('quality' AND 'assessment') OR 'data quality'/exp OR 'data quality' OR ('data' AND 'quality') OR 'consistency' OR 'validity' OR 'efficacy' OR 'completeness' OR 'timeliness' OR 'acceptability' OR 'tool' OR

'instrument' OR 'barriers' OR 'barrier' OR 'facilitators' OR 'facilitator' OR 'bottleneck' OR 'bottlenecks' OR 'limitation' OR 'limitations')

Web of Science	(TS=(newborn OR newborns OR neonate OR neonates OR infant OR infants OR baby OR babies OR “maternal child health care” OR “maternal child health service” OR “maternal child health services” OR “maternal child health center” OR “maternal child health centers”))<br>AND<br>(TS=(africa OR african OR "developing country" OR "developing countries" OR "low income country" OR "low income countries" OR "low income setting" OR "middle income country" OR "middle income countries" OR "middle income setting" OR "low and middle income countries" OR lmic OR afghanistan OR “guinea-bissau” OR "sierra leone" OR benin OR haiti OR somalia OR "burkina faso" OR "north korea" OR "republic of korea" OR "south sudan" OR burundi OR liberia OR "syrian arab republic" OR syria OR "central african republic" OR madagascar OR tajikistan OR chad OR malawi OR tanzania OR "republic of congo" OR "democratic republic of the congo" OR congo OR mali OR togo OR eritrea OR mozambique OR uganda OR ethiopia OR nepal OR yemen OR "republic of yemen" OR gambia OR niger OR guinea OR rwanda OR angola OR india OR "papua new guinea" OR bangladesh OR indonesia OR philippines OR bhutan OR kenya OR "sao tome and principe" OR bolivia OR kiribati OR senegal OR "cabo verde" OR kyrgyzstan OR "kyrgyz republic" OR "solomon islands" OR melanesia OR cambodia OR laos OR "lao people" OR sudan OR cameroon OR lesotho OR "timor-leste" OR comoros OR mauritania OR tunisia OR "democratic republic congo" OR "congo-brazzaville" OR congo OR micronesia OR ukraina OR "cote ivoire" OR moldova OR uzbekistan OR djibouti OR mongolia OR vanuatu OR egypt OR morocco OR vietnam OR "el salvador" OR myanmar OR "west bank and gaza" OR "west bank" OR gaza OR eswatini OR swaziland OR nicaragua OR zambia OR ghana OR nigeria OR zimbabwe OR honduras OR pakistan) OR TS=(cote AND ivoire))<br>AND<br>(TS=(“Health Facilities” OR “facility-level” OR “facility level” OR facility OR “hospital-level” OR “hospital level” OR hospital OR hospitals OR “centre-level” OR “centre level” OR centre OR center OR “center-level” OR “center level” OR “primary health care unit” OR clinic OR “ambulatory care facility” OR “birthing centre” OR “birthing center” OR nursery))<br>AND<br>(TS=(("quality indicator" OR "quality indicators") AND ("delivery of health care" OR ("delivery" AND health AND "care") OR "healthcare" OR "health care")) OR TS=("health status indicators" OR "health status indicator" OR "health indicator" OR "health indicator" "health indicators" OR "benchmarking" OR "indicators" OR "health metrics" OR "measure" OR "measures" OR "data" OR "data sources" OR "primary data sources" OR "health information systems" OR "health information system" OR "hospital information system" OR "HIS" OR "routine health information" OR "routine health system data" OR "routine health

information system" OR "RHIS" OR "health facility data" OR "routine facility data" OR "health management information system" OR "HMIS" OR "Medical Records" OR "patient medical records" OR "patient records" OR "medical records" OR "administrative data" OR "hospital registries" OR "DHIS" OR "DHIS2" OR "registries" OR "registry" OR "register" OR "registers"))

AND

(TS=(“availability” OR "data accuracy" OR “accuracy” OR “quality” OR ("quality assessment" OR (“quality” AND “assessment”)) OR ("data quality" OR (“data” AND “quality”)) OR “consistency” OR “validity” OR “efficacy” OR “completeness” OR “timeliness” OR “acceptability” OR “tool” OR “instrument” OR “barriers” OR “barrier” OR “facilitators” OR “facilitator” OR “bottleneck” OR “bottlenecks” OR “limitation” OR “limitations”))

(mh:"infant,newborn" OR mh:"maternal-child health services" OR mh:"maternal-child health centers" OR tw:neonate OR tw:neonates OR tw:newborn OR tw:newborns OR tw:infant OR tw:infants OR tw:baby OR tw:babies)
AND
(tw:Africa OR tw:African OR tw: LMIC OR tw:"low-income setting" OR tw:"low-resource setting" OR tw:"low-income countries" OR tw:"low-income country" OR tw:"middle-income setting" OR tw:"middle-resource setting" OR tw:"middle-income countries" OR tw:"middle-income country" OR tw:"low and middle income countries" OR tw:Afghanistan OR tw:"guinea-bissau" OR tw:"sierra leone" OR tw:benin OR tw:Haiti OR tw:Somalia OR tw:"Burkina Faso" OR tw:"Democratic People's Republic of Korea" OR tw:"North Korea" OR tw:"south sudan" OR tw:burundi OR tw:liberia OR tw:syria OR tw:"syrian arab republic" OR tw:"Central African Republic" OR tw:madagascar OR tw:Tajikistan OR tw:Chad OR tw:Malawi OR tw:Tanzania OR tw:"Democratic Republic of the Congo" OR tw:Mali OR tw:Togo OR tw:Eritrea OR tw:Mozambique OR tw:Uganda OR tw:Ethiopia OR tw:Nepal OR tw:Yemen OR tw:"The Gambia" OR tw:Gambia OR tw:Niger OR tw:Guinea OR tw:Rwanda OR tw:Angola OR tw:India OR tw:"Papua New Guinea" OR tw:Bangladesh OR tw:Indonesia OR tw:Philippines OR tw:Bhutan OR tw:Kenya OR tw:"Sao Tome and Principe" OR tw:Bolivia OR tw:Micronesia OR tw:Kiribati OR tw:Senegal OR tw:"Cabo Verde" OR tw:Kyrgyzstan OR tw:"Kyrgyz Republic" OR tw:"Solomon Islands" OR tw:Melanesia OR tw:Cambodia OR tw:"Lao People's Democratic Republic" OR tw:Laos OR tw:Sudan OR tw:Cameroon OR tw:Lesotho OR tw:"Timor-Leste" OR tw:Comoros OR tw:Mauritania OR tw:Tunisia OR tw:"Congo-Brazzaville" OR tw:Congo OR tw:Micronesia OR tw:"Federated States of Micronesia" OR tw:Ukraine OR tw:"Cote d'Ivoire" OR tw:Moldova OR tw:Uzbekistan OR tw:Djibouti OR tw:Mongolia OR tw:Vanuatu OR tw:Egypt OR tw:"Arab Republic of Egypt" OR tw:Morocco OR tw:Vietnam OR tw:"El Salvador" OR tw:Myanmar OR tw:"West Bank and Gaza" OR tw:Eswatini OR tw:Swaziland OR tw:Nicaragua OR tw:Zambia OR tw:Ghana OR tw:Nigeria OR tw:Zimbabwe OR tw:Honduras OR tw:Pakistan)
AND
(mh:"health facilities" OR tw:"facility-level" OR tw:facility OR tw:"facility level" OR tw:"hospital-level" OR tw:"hospital level" OR tw:hospital OR tw:"centre-level" OR tw:"centre level" OR tw:centre OR tw:"primary health care unit" OR tw:clinic OR tw:"ambulatory care facility" OR tw:"birthing centre" OR tw:nursery)
AND (mh:"quality indicators, health care" OR (tw:"quality indicators"
AND
((mh:"delivery of health care" OR (tw:delivery AND tw:health AND tw:care) OR tw:"delivery of health care" OR tw:healthcare) OR tw:"health care")) OR tw:"health status indicators" OR tw:"health indicators"

Global Index Medicus

OR tw:benchmarking OR tw:indicator OR tw:indicators OR tw:"health metrics" OR tw:measure OR

tw:measures OR tw:data OR tw:"data sources" OR tw:"primary data sources" OR tw:"health information systems" OR tw:"health information system" OR tw:"hospital information systems" OR tw:HIS OR tw:"routine health information" OR tw:"routine health data" OR tw:"routine health information system" OR tw:RHIS OR tw:"health facility data" OR tw:"routine facility data" OR tw:"health management information system" OR tw:HMIS OR tw:"medical records" OR tw:"patient medical records" OR tw:"patient records" OR tw:"medical records" OR tw:"administrative data" OR tw:"hospital registries" OR tw:DHIS OR tw:DHIS2 OR tw:registries OR tw:registry OR tw:register OR tw:registers)
AND
(tw:availability OR tw:"data accuracy" OR tw:accuracy OR tw:quality OR (tw:"quality assessment" OR (tw:quality AND tw:assessment)) OR (tw:"data quality" OR(tw:data AND tw:quality)) OR tw:consistency OR tw:validity OR tw:efficacy OR tw:completeness OR tw:timeliness OR tw:acceptability OR tw:tool OR tw:instrument OR tw:barriers OR tw: barrier OR tw:facilitators OR tw:facilitator OR tw:bottleneck OR tw:bottlenecks OR tw:limitation OR tw: limitations)

([mh "infant, newborn"] OR [mh "maternal-child health services"] OR [mh "maternal-child health centers"] OR neonate OR neonates OR newborn OR newborns OR infant OR infants OR baby OR babies)
AND
([mh Africa] OR Africa OR African OR LMIC OR "low-income setting" OR "low-resource setting" OR "low-income countries" OR "low-income country" OR "middle-income setting" OR "middle-resource setting" OR "middle-income countries" OR "middle-income country" OR "low and middle income countries" OR [mh Afghanistan] OR [mh "guinea-bissau"] OR [mh "sierra leone"] OR [mh benin] OR [mh Haiti] OR [mh Somalia] OR [mh "Burkina Faso"] OR [mh "Democratic People's Republic of Korea"] OR "North Korea" OR [mh "south sudan"] OR [mh Burundi] OR [mh Liberia] OR [mh Syria] OR "syrian arab republic" OR [mh "Central African Republic"] OR [mh Madagascar] OR [mh Tajikistan] OR [mh Chad] OR [mh Malawi] OR [mh Tanzania] OR [mh "Democratic Republic of the Congo"] OR [mh Mali] OR [mh Togo] OR [mh Eritrea] OR [mh Mozambique] OR [mh Uganda] OR [mh Ethiopia] OR [mh Nepal] OR [mh Yemen] OR "The Gambia" OR [mh Gambia] OR [mh Niger] OR [mh Guinea] OR [mh Rwanda] OR [mh Angola] OR [mh India] OR [mh "Papua New Guinea"] OR [mh Bangladesh] OR [mh Indonesia] OR [mh Philippines] OR [mh Bhutan] OR [mh Kenya] OR [mh "Sao Tome and Principe"] OR [mh Bolivia] OR [mh Micronesia] OR [mh Kiribati] OR [mh Senegal] OR [mh "Cabo Verde"] OR [mh Kyrgyzstan] OR "Kyrgyz Republic" OR [mh "Solomon Islands"] OR [mh Melanesia] OR [mh Cambodia] OR "Lao People's Democratic Republic" OR [mh Laos] OR [mh Sudan] OR [mh Cameroon] OR [mh Lesotho] OR [mh "Timor-Leste"] OR [mh Comoros] OR [mh Mauritania] OR [mh Tunisia] OR "Congo-Brazzaville" OR [mh Congo] OR [mh Micronesia] OR "Federated States of Micronesia" OR [mh Ukraine] OR [mh "Cote d'Ivoire"] OR [mh Moldova] OR [mh Uzbekistan] OR [mh Djibouti] OR [mh Mongolia] OR [mh Vanuatu] OR [mh Egypt] OR "Arab Republic of Egypt" OR [mh Morocco] OR [mh Vietnam] OR [mh "El Salvador"] OR [mh Myanmar] OR [mh "West Bank and Gaza"] OR [mh Eswatini] OR [mh Swaziland] OR [mh Nicaragua] OR [mh Zambia] OR [mh Ghana] OR [mh Nigeria] OR [mh Zimbabwe] OR [mh Honduras] OR [mh Pakistan])
AND
([mh “Health Facilities”] OR “facility-level” OR “facility level” OR facility OR “hospital-level” OR “hospital level” OR hospital OR hospitals OR “centre-level” OR “centre level” OR centre OR center OR “center-level” OR “center level” OR “primary health care unit” OR clinic OR “ambulatory care facility” OR “birthing centre” OR “birthing center” OR nursery)
AND

Cochrane Library

([mh "quality indicators, health care"] OR ("quality indicators" AND (([mh "delivery of health care"] OR (delivery AND health AND care) OR “delivery of health care” OR healthcare) OR "health care")) OR [mh "health status indicators"] OR "health status indicator" OR "health indicator" OR "health indicator" OR

"health indicators" OR [mh benchmarking] OR indicators OR "health metrics" OR measure OR measures OR data OR "data sources" OR "primary data sources" OR [mh "health information systems"] OR "health information system" OR "hospital information system" OR HIS OR "routine health information" OR "routine health system data" OR "routine health information system" OR RHIS OR "health facility data" OR "routine facility data" OR "health management information system" OR HMIS OR [mh "Medical Records"] OR "patient medical records" OR "patient records" OR "medical records" OR "administrative data" OR "hospital registries" OR DHIS OR DHIS2 OR [mh registries] OR registry OR register OR registers)
AND
("availability" OR [mh "data accuracy"] OR accuracy OR quality OR ("quality assessment" OR (quality AND assessment)) OR ("data quality" OR (data AND quality)) OR consistency OR validity OR efficacy OR completeness OR timeliness OR acceptability OR tool OR instrument OR barriers OR barrier OR facilitators OR facilitator OR bottleneck OR bottlenecks OR limitation OR limitations)

PubMed	("infant, newborn"[MeSH Terms] OR "maternal-child health services"[MeSH Terms] OR "maternal-child health centers"[MeSH Terms] OR “neonate”[All Fields] OR “neonates”[All Fields] OR “newborn”[All Fields] OR “newborns”[All Fields] OR “infant”[All Fields] OR “infants”[All Fields] OR “baby”[All Fields] OR “babies”[All Fields]) AND (“Africa”[MeSH Terms] OR “Africa”[All Fields] OR “African”[All Fields] OR “LMIC”[All Fields] OR "low-income setting"[All Fields] OR "low-resource setting"[All Fields] OR "low-income countries"[All Fields] OR "low-income country"[All Fields] OR "middle-income setting"[All Fields] OR "middle-resource setting"[All Fields] OR "middle-income countries"[All Fields] OR "middle-income country"[All Fields] OR "low and middle income countries"[All Fields] OR ("Afghanistan"[MeSH Terms] OR "Afghanistan"[All Fields]) OR ("Guinea-Bissau"[MeSH Terms] OR "Guinea-Bissau"[All Fields]) OR ("Sierra Leone"[MeSH Terms] OR "Sierra Leone"[All Fields]) OR ("Benin"[MeSH Terms] OR "Benin"[All Fields]) OR ("Haiti"[MeSH Terms] OR "Haiti"[All Fields]) OR ("Somalia"[MeSH Terms] OR "Somalia"[All Fields]) OR (“Burkina Faso"[MeSH Terms] OR "Burkina Faso"[All Fields]) OR ("Democratic People's Republic of Korea"[MeSH Terms] OR "Democratic People's Republic of Korea"[All Fields] OR "North Korea"[All Fields]) OR (“South Sudan”[MeSH Terms] OR "South Sudan"[All Fields]) OR ("Burundi"[MeSH Terms] OR "Burundi"[All Fields]) OR ("Liberia"[MeSH Terms] OR "Liberia"[All Fields]) OR ("Syrian Arab Republic"[All Fields] OR "Syria"[MeSH Terms] OR "Syria"[All Fields]) OR (“Central African Republic”[MeSH Terms] OR "Central African Republic"[All Fields]) OR ("Madagascar"[MeSH Terms] OR "Madagascar"[All Fields]) OR ("Tajikistan"[MeSH Terms] OR "Tajikistan"[All Fields]) OR ("Chad"[MeSH Terms] OR "Chad"[All Fields]) OR ("Malawi"[MeSH Terms] OR "Malawi"[All Fields]) OR ("Tanzania"[MeSH Terms] OR "Tanzania"[All Fields]) OR (“Democratic Republic of the Congo”[MeSH Terms] OR "Democratic Republic of the Congo"[All Fields]) OR ("Mali"[MeSH Terms] OR "Mali"[All Fields]) OR ("Togo"[MeSH Terms] OR "Togo"[All Fields]) OR ("Eritrea"[MeSH Terms] OR "Eritrea"[All Fields]) OR ("Mozambique"[MeSH Terms] OR "Mozambique"[All Fields]) OR ("Uganda"[MeSH Terms] OR "Uganda"[All Fields]) OR ("Ethiopia"[MeSH Terms] OR "Ethiopia"[All Fields]) OR ("Nepal"[MeSH Terms] OR "Nepal"[All Fields]) OR ("Republic of Yemen"[All Fields] OR "Yemen"[MeSH Terms] OR "Yemen"[All Fields]) OR ("The Gambia"[All Fields] OR “Gambia”[MeSH Terms] OR “Gambia”[All Fields]) OR ("Niger"[MeSH Terms] OR "Niger"[All Fields]) OR ("Guinea"[MeSH Terms] OR "Guinea"[All Fields]) OR ("Rwanda"[MeSH Terms] OR "Rwanda"[All Fields]) OR ("Angola"[MeSH Terms] OR "Angola"[All Fields]) OR ("India"[MeSH Terms] OR "India"[All Fields]) OR ("Papua New Guinea"[MeSH Terms] OR “Papua New Guinea”[All Fields]) OR ("Bangladesh"[MeSH Terms] OR "Bangladesh"[All Fields]) OR ("Indonesia"[MeSH Terms] OR "Indonesia"[All Fields]) OR ("Philippines"[MeSH Terms] OR "Philippines"[All Fields]) OR ("Bhutan"[MeSH Terms] OR "Bhutan"[All Fields]) OR ("Kenya"[MeSH Terms] OR "Kenya"[All Fields]) OR ("Sao Tome and Principe"[MeSH Terms] OR “Sao Tome and Principe”[All Fields]) OR ("Bolivia"[MeSH Terms] OR "Bolivia"[All Fields]) OR

("Micronesia"[MeSH Terms] OR "Micronesia"[All Fields] OR "Kiribati"[All Fields]) OR ("Senegal"[MeSH Terms] OR "Senegal"[All Fields]) OR ("Cabo Verde"[MeSH Terms]OR “Cabo Verde”[All Fields]) OR ("Kyrgyz Republic"[All Fields] OR "Kyrgyzstan"[MeSH Terms] OR "Kyrgyzstan"[All Fields]) OR ("Solomon Islands"[All Fields] OR “Melanesia”[MeSH Terms]) OR ("Cambodia"[MeSH Terms] OR "Cambodia"[All Fields]) OR ("Lao People's Democratic Republic"[All Fields] OR "Laos"[MeSH Terms] OR "Laos"[All Fields]) OR ("Sudan"[MeSH Terms] OR "Sudan"[All Fields]) OR ("Cameroon"[MeSH Terms] OR "Cameroon"[All Fields]) OR ("Lesotho"[MeSH Terms] OR "Lesotho"[All Fields]) OR ("Timor-Leste"[MeSH Terms] OR “Timor-Leste”[All Fields]) OR ("Comoros"[MeSH Terms] OR "Comoros"[All Fields]) OR ("Mauritania"[MeSH Terms] OR "Mauritania"[All Fields]) OR ("Tunisia"[MeSH Terms] OR "Tunisia"[All Fields]) OR ("Democratic Republic of the Congo"[MeSH Terms] OR “Democratic Republic of the Congo”[All Fields]) OR ("Congo-Brazzaville"[All Fields] OR “Congo”[MeSH Terms] OR “Congo”[All Fields]) OR ("Federated States of Micronesia"[All Fields] OR "Micronesia"[MeSH Terms] OR "Micronesia"[All Fields]) OR ("Ukraine"[MeSH Terms] OR "Ukraine"[All Fields]) OR ("Cote d'Ivoire"[MeSH Terms] OR “Cote d’Ivoire”[All Fields]) OR ("Moldova"[MeSH Terms] OR "Moldova"[All Fields]) OR ("Uzbekistan"[MeSH Terms] OR "Uzbekistan"[All Fields]) OR ("Djibouti"[MeSH Terms] OR "Djibouti"[All Fields]) OR ("Mongolia"[MeSH Terms] OR "Mongolia"[All Fields]) OR ("Vanuatu"[MeSH Terms] OR "Vanuatu"[All Fields]) OR ("Arab Republic of Egypt"[All Fields] OR "Egypt"[MeSH Terms] OR "Egypt"[All Fields]) OR ("Morocco"[MeSH Terms] OR "Morocco"[All Fields]) OR ("Vietnam"[MeSH Terms] OR "Vietnam"[All Fields]) OR ("El Salvador"[MeSH Terms] OR “El Salvador”[All Fields]) OR ("Myanmar"[MeSH Terms] OR "Myanmar"[All Fields]) OR ("West Bank and Gaza"[All Fields]) OR ("Eswatini"[MeSH Terms] OR "Eswatini"[All Fields] OR "Swaziland"[All Fields]) OR ("Nicaragua"[MeSH Terms] OR "Nicaragua"[All Fields]) OR ("Zambia"[MeSH Terms] OR "Zambia"[All Fields]) OR ("Ghana"[MeSH Terms] OR "Ghana"[All Fields]) OR ("Nigeria"[MeSH Terms] OR "Nigeria"[All Fields]) OR ("Zimbabwe"[MeSH Terms] OR "Zimbabwe"[All Fields]) OR ("Honduras"[MeSH Terms] OR "Honduras"[All Fields]) OR ("Pakistan"[MeSH Terms] OR "Pakistan"[All Fields])) AND (“Health Facilities”[MeSH Terms] OR "facility-level"[All Fields] OR "facility level"[All Fields] OR "facility"[All Fields] OR "hospital-level"[All Fields] OR "hospital level"[All Fields] OR "hospital"[All Fields] OR "centre-level"[All Fields] OR "centre level"[All Fields] OR "centre"[All Fields] OR "center"[All Fields] OR "center-level"[All Fields] OR "center level"[All Fields] OR "primary health care unit"[All Fields] OR “clinic” [All Fields] OR “ambulatory care facility”[All Fields] OR “birthing centre”[All Fields] OR “birthing center”[All Fields] OR “nursery”[All Fields]) AND ("quality indicators, health care"[MeSH Terms] OR ("quality indicators"[All Fields] AND (("delivery of health care"[MeSH Terms] OR ("delivery"[All Fields] AND "health"[All Fields] AND "care"[All Fields]) OR "delivery of health care"[All Fields] OR "healthcare"[All Fields]) OR "health care"[All Fields])) OR "health status indicators"[MeSH Terms] OR "health status indicators"[All Fields] OR

"health indicators"[All Fields] OR "benchmarking"[MeSH Terms] OR "benchmarking"[All Fields] OR "indicator"[All Fields] OR "indicators"[All Fields] OR "health metrics"[All Fields] OR "measure"[All Fields] OR "measures"[All Fields] OR "data"[All Fields] OR "data sources"[All Fields] OR "primary data sources"[All Fields] OR "health information systems"[MeSH Terms] OR "health information system"[All Fields] OR "hospital information system"[All Fields] OR "HIS"[All Fields] OR "routine health information"[All Fields] OR "routine health system data"[All Fields] OR "routine health information system"[All Fields] OR "RHIS"[All Fields] OR "health facility data"[All Fields] OR "routine facility data"[All Fields] OR "health management information system"[All Fields] OR "HMIS"[All Fields] OR "Medical Records"[MeSH Terms] OR "patient medical records"[All Fields] OR "patient records"[All Fields] OR "medical records"[All Fields] OR "administrative data"[All Fields] OR "hospital registries"[All Fields] OR "DHIS"[All Fields] OR "DHIS2"[All Fields] OR "Registries"[MeSH Terms] OR "registries"[All Fields] OR "registry"[All Fields] OR "register"[All Fields] OR "registers"[All Fields]) AND ("availability"[All Fields] OR "data accuracy"[MeSH Terms] OR "accuracy"[All Fields] OR "quality"[All Fields] OR ("quality assessment"[All Fields] OR ("quality"[All Fields] AND "assessment"[All Fields])) OR ("data quality"[All Fields] OR ("data"[All Fields] AND "quality"[All Fields])) OR "consistency"[All Fields] OR "validity"[All Fields] OR "efficacy"[All Fields] OR "completeness"[All Fields] OR "timeliness"[All Fields] OR "acceptability"[All Fields] OR "tool"[All Fields] OR "instrument"[All Fields] OR "barriers"[All Fields] OR "barrier"[All Fields] OR "facilitators"[All Fields] OR "facilitator"[All Fields] OR "bottleneck"[All Fields] OR "bottlenecks"[All Fields] OR "limitation"[All Fields] OR "limitations"[All Fields])

Table S4: Characteristics of included studies

Author, Year	Study design	Country/ies	Health facility type/s	Health facility description (n)	Health facility sampling criteria	Data source/s	Eligible
Chiba, 2012	Cross-sectional	Kenya	public	rural district hospitals (2)	-	- childbirth register<br>- monthly workload reports	women<br>particip<br>(Hospita<br>deliverie<br>- 1806 d
Day, 2020a (Gore-Langton)	Cross-sectional	Bangladesh<br>Nepal<br>Tanzania	public	hospitals (5)	implementing selected MNH interventions	labour ward register	neonate<br>particip<br>(20,075)
Bhattacharya, 2019a (Allen)	Cross-sectional	Nigeria	public	primary health facilities (10)	with highest birth volumes 6 months prior to data collection	labour ward register	neonate<br>particip<br>(average
Bhattacharya, 2019b (Umar)	Cross-sectional	Nigeria	public	- primary health facilities (97) - referral facilities (18)	-	labour ward register	neonate<br>particip
Sharma, 2016	Cross-sectional	India	public	sub-centres (209)	sub-centre areas randomly selected	childbirth register	neonate<br>particip
Lambo, 2011	Cross-sectional	Pakistan	public, private	- government hospitals (7)<br>- private pediatric clinics (5)	had not achieved maternal and neonatal tetanus elimination in Dec 2010	- vital event, inpatient, and outpatient registers<br>- medical records	neonate<br>particip

				- tertiary referral hospitals (5)			
Day, 2020 b (Rahman)	Cross-sectional	Bangladesh<br>Nepal<br>Tanzania	public	hospitals (5)	providing CEmONC	labour ward register	neonate<br>participa
Hazard, 2017	Cross-sectional	Bangladesh	not clear	- secondary hospital (1)<br>- primary level Upazila health complex (7)<br>- hospital (1)<br>- tertiary medical college hospital (6)	-	medical records	neonate<br>participa
Kayode, 2014	Cross-sectional	Ghana	not clear	- district hospital, secondary level (7)<br>- polyclinic/health center, primary level (6)	-	labour ward register	neonate<br>participa
Kihuba, 2014	Cross-sectional	Kenya	public	public hospitals, internship training centers (22)		labour ward register	neonate<br>participa
Landry, 2014	Cross-sectional	Bangladesh<br>Guinea<br>Mali<br>Niger<br>Uganda	public, private	-urban government facilities (6) - rural faith-based facilities (1)<br>- rural private facilities (2)	-	medical records	neonate<br>participa

Miller, 2020	Quasi-experimental	Kenya Uganda	public, private	- district referral facilities (3) - facilities (15) - regional referral hospitals (1) - missionary district facilities (2) - missionary facilities (2)	>= 200 annual births	labour ward register	neonate[illegible] particip[illegible]
Mony, 2015	Cohort	India	not clear	- district hospitals (9) - sub-district hospital (1) - community health centers (11)	implementing SCC with high-volume deliveries	labour ward register	neonate[illegible] particip[illegible]
Nyamtema, 2008	Cross-sectional	Tanzania	public	hospitals (4)	all in Dar es Salam	medical records	neonate[illegible] particip[illegible]
Phillips, 2019	Cross-sectional	India	public	hospitals (40)	10 hospitals in 4 districts with highest patient loads in Uttar Pradesh	- medical records - labour ward register	neonate[illegible] particip[illegible]
Plotkin, 2018	Cross-sectional	Tanzania	public	health facilities (10)	underwent refresher training on perinatal death classification and use of handheld Doppler devices to assess fetal heart rate on admission to maternity services	labour ward register	neonate[illegible] particip[illegible]
Sychareun, 2014	Cross-sectional	Lao PDR	not clear	- hospitals, health centers (16) - district hospitals (109)	in regions implementing integrated MNCH program with MoH and WHO	labour ward register	neonate[illegible] particip[illegible]
Kong, 2020	Cross-sectional	Bangladesh Nepal Tanzania	public	hospitals (5)	providing CEmONC	labour ward register	neonate[illegible] particip[illegible]

Tahsina, 2021	Cross-sectional	Bangladesh<br>Nepal<br>Tanzania	public	hospitals (5)	providing CEmONC	labour ward register	neonate<br>particip
Salim, 2021	Cross-sectional	Bangladesh<br>Nepal<br>Tanzania	public	hospitals (5)	providing CEmONC	KMC<br>ward/corner/programme<br>register	neonate<br>particip
Rahman, 2021	Cross-sectional	Bangladesh<br>Nepal<br>Tanzania	public	hospitals (5)	providing CEmONC	medical records	neonate<br>particip
Kc, 2021	Cross-sectional	Bangladesh<br>Nepal<br>Tanzania	public	hospitals (5)	providing CEmONC	labour ward register	neonate<br>particip
Peven, 2021	Cross-sectional	Bangladesh<br>Nepal<br>Tanzania	public	hospitals (5)	providing CEmONC	labour ward register	neonate<br>particip
Zaman, 2021	Cross-sectional	Bangladesh<br>Nepal<br>Tanzania	public	hospitals (5)	providing CEmONC	labour ward register	neonate<br>particip
Keating, 2019	Quasi-experimental	Kenya<br>Uganda	not<br>clear	- referral hospitals (2)<br>- sub-county hospitals (10)<br>- health centers (4)<br>- mission hospitals (5)<br>- district hospitals (2)	baseline measures from<br>centers participating in<br>neonatal data quality<br>improvement initiative	labour ward register	neonate<br>particip

Gebrehiwot, 2020	Cross-sectional	Ethiopia	public, private	- public hospitals, health centers, and clinics (2545)<br>- NGO hospitals, health centers, and clinics (14)<br>- private for-profit hospitals, centres, and clinics (23)<br>- mission/faith based hospitals, health centres, and clinics (29)	using partogram	medical records	neonate [illegible]<br>particip[illegible]
Kc, 2020	Nested observational study	Nepal	public	public hospitals (12)	providing referral level obstetric and neonatal care	medical records	neonate [illegible]<br>particip[illegible]
Bhattacharya, 2020	Quasi-experimental	Nigeria	not clear	- primary facilities (97)	randomly sampled	labour ward register	neonate [illegible]<br>particip[illegible]
Fawole, 2007	Cross-sectional	Nigeria	public	university hospital (1)	-	medical records	neonate [illegible]<br>particip[illegible]
Ndira, 2008	Quasi-experimental	Uganda	public	district hospital (1)	-	- medical records<br>- labour ward register	neonate [illegible]<br>particip[illegible]

Nicol, 2016	Cross-sectional	South Africa	public	- public hospitals (6)<br>- primary health centers (2)<br>- clinics (34)<br>- gateway clinics (3)<br>- midwife obstetric units (3) - community health centers (2) - community day centers (7)	with health information system for routine PMTCT indicators, provide PMTCT and see women and children, operational for full study period	PMTCT register	neonate[illegible]<br>particip[illegible]
Duffy, 2009	Cross-sectional	Ethiopia	private	church-affiliated hospital (1)	-	labour ward register	neonate[illegible]<br>particip[illegible]
Broughton, 2013	Cross-sectional	Afghanistan	public, private	- maternity teaching hospital (1) - maternity ward of general teaching hospital (1)<br>- maternity ward of provincial hospital (1)	-	- medical records<br>- labour ward register	neonate[illegible]<br>particip[illegible]
Mate, 2009	Cross-sectional	South Africa	public	- clinics (86)<br>- hospitals (13)	participating in PMTCT service quality systems strengthening program	registers	neonate[illegible]<br>particip[illegible]

Table S5: Estimates of timeliness and completeness

**Included in figure 3**			
**Author, year**	**Data source**	**Measure**	**Point estimate**
**Completeness in case notes**			
Gebrehiwot, 2020	Case notes	Mode of delivery (% mode of delivery recorded in partographs)	95.[illegible]
Rahman, 2021	Case notes	Discharge condition (% baby's condition at discharge recorded in Muhimbili, Tanzania)	10[illegible]
Rahman, 2021	Case notes	Discharge condition (% baby's condition at discharge recorded in Azimpur, Bangladesh)	10[illegible]
Rahman, 2021	Case notes	Discharge condition (% baby's condition at discharge recorded in Temeke, Tanzania)	94.[illegible]

Rahman, 2021	Case notes	Discharge condition (% baby's condition at discharge recorded in Kushtia, Bangladesh)	90.[illegible]
Rahman, 2021	Case notes	Discharge condition (% baby's condition at discharge recorded in Pokhara, Nepal)	99.[illegible]
Nyamtema, 2008	Case notes	Fetal heart rate (% partograms with fetal heart rate recorded)	8[illegible]
Fawole, 2007	Case notes	Fetal heart rate (% partograms with regular fetal heart rate recorded)	93.[illegible]
Gebrehiwot, 2020	Case notes	Fetal heart rate (% partograms with fetal heart rate recorded every 30 minutes)	95.[illegible]
Landry, 2014	Case notes	Time/date/place of delivery (% records with time of c-section delivery in Mali)	75.[illegible]
Landry, 2014	Case notes	Time/date/place of delivery (% records with time of c-section delivery in hospital 1 in Guinea)	72.[illegible]
Landry, 2014	Case notes	Time/date/place of delivery (% records with time of c-section delivery in hospital 1 in Niger)	96.[illegible]
Landry, 2014	Case notes	Time/date/place of delivery (% records with time of c-section delivery in hospital 3 in Niger)	98.[illegible]
Landry, 2014	Case notes	Time/date/place of delivery (% records with time of c-section delivery in hospital 2 in Niger)	91.[illegible]
Landry, 2014	Case notes	Time/date/place of delivery (% records with time of c-section delivery in Uganda)	8[illegible]
Landry, 2014	Case notes	Time/date/place of delivery (% records with time of c-section delivery in Bangladesh)	99.[illegible]
Landry, 2014	Case notes	Time/date/place of delivery (% records with time of c-section delivery in hospital 2 in Guinea)	6[illegible]
Gebrehiwot, 2020	Case notes	Time/date/place of delivery (% partograms with time of delivery recorded)	84.[illegible]
Sharma, 2016	Case notes	Time/date/place (% records with date of delivery)	94.[illegible]
Sharma, 2016	Case notes	Time/date/place (% records with place of delivery)	93.[illegible]
Landry, 2014	Case notes	Birth outcome (% c-section records with information on birth outcome in Mali)	10[illegible]
Landry, 2014	Case notes	Birth outcome (% c-section records with information on birth outcome in hospital 1 Guinea)	97.[illegible]
Landry, 2014	Case notes	Birth outcome (% c-section records with information on birth outcome in hospital 1 Niger)	99.[illegible]
Landry, 2014	Case notes	Birth outcome (% c-section records with information on birth outcome in hospital 3 Niger)	99.[illegible]
Landry, 2014	Case notes	Birth outcome (% c-section records with information on birth outcome in hospital 1 Uganda)	89.[illegible]
Landry, 2014	Case notes	Birth outcome (% c-section records with information on birth outcome in hospital 2 Niger)	99.[illegible]
Landry, 2014	Case notes	Birth outcome (% c-section records with information on birth outcome in hospital 2 Niger)	59.[illegible]
Landry, 2014	Case notes	Birth outcome (% c-section records with information on birth outcome in Bangladesh)	8[illegible]
Nyamtema, 2008	Case notes	Birth outcome (% partograms with condition at birth)	5[illegible]
Landry, 2014	Case notes	Birth outcome (% records with information on birth outcome in hospital 2 Guinea)	96.[illegible]
Sharma, 2016	Case notes	Birth outcome (% records with information on birth outcome)	93.[illegible]
Landry, 2014	Case notes	Time of death (% records with time of perinatal death among c-sections in Mali)	6[illegible]
Landry, 2014	Case notes	Time of death (% records with time of perinatal death among c-sections in hospital 1 in Guinea)	98.[illegible]
Landry, 2014	Case notes	Time of death (% records with time of perinatal death among c-sections in hospital 1 in Niger)	85.[illegible]

Landry, 2014	Case notes	Time of death (% records with time of perinatal death among c-sections in hospital 3 in Niger)	23.[illegible]
Landry, 2014	Case notes	Time of death (% records with time of perinatal death among c-sections in hospital 1 in Uganda)	88.[illegible]
Landry, 2014	Case notes	Time of death (% records with time of perinatal death among c-sections in hospital 2 in Niger)	4[illegible]
Landry, 2014	Case notes	Time of death (% records with time of perinatal death among c-sections in hospital 2 in Uganda)	10[illegible]
Landry, 2014	Case notes	Time of death (% records with time of perinatal death among c-sections in Bangladesh)	
Landry, 2014	Case notes	Time of death (% records with time of perinatal death among c-sections in hospital 2 in Guinea)	98.[illegible]
**Completeness in register entries**			
Salim, 2021	Register entries	KMC (% KMC recorded)	99.[illegible]
Day, 2020b (Rahman)	Register entries	KMC (% KMC initiation recorded)	99.[illegible]
Zaman, 2021	Register entries	Cord care (% chlorhexidine application recorded among all live births)	94.[illegible]
Zaman, 2021	Register entries	Cord care (% chlorhexidine application recorded among vaginal births)	95.[illegible]
Zaman, 2021	Register entries	Cord care (% chlorhexidine application recorded among c-section births)	87.[illegible]
Bhattacharya, 2019a (Allen)	Register entries	Gestational age (% preterm birth recorded)	7[illegible]
Day, 2020a (Gore)	Register entries	Gestational age (% gestational age recorded in Muhimbili, Tanzania)	96.[illegible]
Day, 2020a (Gore)	Register entries	Gestational age (% gestational age recorded in Pokhara, Nepal)	95.[illegible]
Miller, 2020	Register entries	Gestational age (% gestational age recorded)	77.[illegible]
Chiba, 2012	Register entries	Time/date/place of delivery (% date of delivery recorded in Bondo hospital)	73.[illegible]
Chiba, 2012	Register entries	Time/date/place of delivery (% date of delivery recorded in Siaya hospital)	99.[illegible]
Chiba, 2012	Register entries	Mode of delivery (% mode of delivery recorded at Bondo hospital)	68.[illegible]
Day, 2020a (Gore)	Register entries	Mode of delivery (% mode of delivery recorded at Siaya hospital)	99.[illegible]
Day, 2020a (Gore)	Register entries	Mode of delivery (% mode of delivery recorded at Muhimbili, Tanzania)	10[illegible]
Day, 2020a (Gore)	Register entries	Mode of delivery (% mode of delivery recorded at Kushtia, Bangladesh)	99.[illegible]
Day, 2020a (Gore)	Register entries	Mode of delivery (% mode of delivery recorded at Temeke, Tanzania)	10[illegible]
Day, 2020a (Gore)	Register entries	Mode of delivery (% mode of delivery recorded at Azimpur, Bangladesh)	10[illegible]
Day, 2020a (Gore)	Register entries	Mode of delivery (% mode of delivery recorded at Pohkara, Nepal)	99.[illegible]
Chiba, 2012	Register entries	Sex (% sex recorded at Bondo hospital)	67.[illegible]
Chiba, 2012	Register entries	Sex (% sex recorded at Siaya hospital)	98.[illegible]
Day, 2020a (Gore)	Register entries	Sex (% sex recorded at Muhimbili, Tanzania)	10[illegible]
Day, 2020a (Gore)	Register entries	Sex (% sex recorded at Kushtia, Bangladesh)	78.[illegible]

Day, 2020a (Gore)	Register entries	Sex (% sex recorded at Temeke, Tanzania)	10[illegible]
Day, 2020a (Gore)	Register entries	Sex (% sex recorded at Azimpur, Bangladesh	99.[illegible]
Day, 2020a (Gore)	Register entries	Sex (% sex recorded at Pokhara, Nepal)	10[illegible]
Miller, 2020	Register entries	Sex (% sex recorded)	91.[illegible]
Chiba, 2012	Register entries	Birthweight (% birthweight recorded at Bondo hospital)	69.[illegible]
Chiba, 2012	Register entries	Birthweight (% birthweight recorded at Siaya hospital)	97.[illegible]
Bhattacharya, 2019a (Allen)	Register entries	Birthweight (% birthweight recorded)	9[illegible]
Day, 2020a (Gore)	Register entries	Birthweight (% birthweight recorded at Muhimbili, Tanzania)	99.[illegible]
Day, 2020a (Gore)	Register entries	Birthweight (% birthweight recorded at Kushtia, Bangladesh)	66.[illegible]
Day, 2020a (Gore)	Register entries	Birthweight (% birthweight recroded at Temeke, Tanzania)	10[illegible]
Day, 2020a (Gore)	Register entries	Birthweight (% birthweight recorded at Azimpur, Bangladesh)	99.[illegible]
Day, 2020a (Gore)	Register entries	Birthweight (% birthweight recorded at Pokhara, Nepal)	99.[illegible]
Miller, 2020	Register entries	Birthweight (% birthweight recorded)	89.[illegible]
Kong, 2020	Register entries	Birthweight (% birthweight recorded)	96.8[illegible]
Chiba, 2012	Register entries	Infant feeding type (% infant feeding type recorded at Bondo hospital)	61.[illegible]
Chiba, 2012	Register entries	Infant feeding type (% infant feeding type recorded at Siaya hospital)	97.[illegible]
Day, 2020a (Gore)	Register entries	Stimulation (% stimulation recorded in Muhumbili, Tanzania)	10[illegible]
Day, 2020a (Gore)	Register entries	Stimulation (% stimulation recorded in Temeke, Tanzania)	10[illegible]
Kc, 2021	Register entries	Stimulation (% stimulation recorded among live births and fresh stillbirths)	34.[illegible]
Day, 2020a (Gore)	Register entries	Bag mask ventilation (% bag mask ventilation recorded in Muhimbili, Tanzania)	10[illegible]
Day, 2020a (Gore)	Register entries	Bag mask ventilation (% bag mask ventilation recorded in Temeke, Tanzania)	10[illegible]
Kc, 2021	Register entries	Bag mask ventilation (% bag mask ventilation recorded among non-breathing infants)	35.[illegible]
Kc, 2021	Register entries	Bag mask ventilation (% bag mask ventilation recorded among non-crying infants)	35.[illegible]
Kc, 2021	Register entries	Bag mask ventilation (% bag mask ventilation recorded among all live births and fresh stillbirths)	3[illegible]
Day, 2020b (Rahman)	Register entries	Bag mask ventilation (% bag mask ventilation recorded)	34.[illegible]
Day, 2020a (Gore)	Register entries	Discharge condition (% condition at discharge recorded in Muhimbili, Tanzania)	10[illegible]
Day, 2020a (Gore)	Register entries	Discharge condition (% condition at discharge recorded in Kushtia, Bangladesh)	70.[illegible]
Day, 2020a (Gore)	Register entries	Discharge condition (% condition at discharge recorded in Temeke, Tanzania)	9[illegible]
Day, 2020a (Gore)	Register entries	Discharge condition (% condition at discharge recorded in Azimpur, Bangladesh)	29.[illegible]

Day, 2020a (Gore)	Register entries	Discharge condition (% condition at discharge recorded in Pokhara, Nepal)	99.
Chiba, 2012	Register entries	Vaccination/prophylactic (% nevirapine taken by baby recorded in Bondo hospital)	47.
Chiba, 2012	Register entries	Vaccination/prophylactic (% nevirapine taken by baby recorded in Siaya hospital)	28.
Sychareun, 2014	Register entries	Early postnatal care (% first prenatal care visit within 7 days of birth recorded)	2
Sychareun, 2014	Register entries	Skilled birth attendant (% skilled birth attendent recorded)	2
Day, 2020a (Gore)	Register entries	Stillbirth type (% stillbirth type (fresh/macerated) recorded in Muhimbili, Tanzania)	10
Day, 2020a (Gore)	Register entries	Stillbirth type (% stillbirth type (fresh/macerated) recorded in Kushtia, Bangladesh)	
Day, 2020a (Gore)	Register entries	Stillbirth type (% stillbirth type (fresh/macerated) recorded in Temeke, Tanzania)	10
Day, 2020a (Gore)	Register entries	Stillbirth type (% stillbirth type (fresh/macerated) recorded in Pokhara, Nepal)	45.
Day, 2020a (Gore)	Register entries	Early initiation of breastfeeding (% breastfeeding within one hour of birth recorded in Muhimbili, Tanzania)	
Day, 2020a (Gore)	Register entries	Early initiation of breastfeeding (% breastfeeding within one hour of birth recorded in Temeke, Tanzania)	99.
Tahsina, 2021	Register entries	Early initiation of breastfeeding (% early initiation of breastfeeding recorded)	92.
Day, 2020b (Rahman)	Register entries	Early initiation of breastfeeding (% breastfeeding within one hour of birth recorded)	92.

Excluded from figure 3

**Author, year**	**Data source**	**Measure**	**Point estimate**
**Completeness for composite data elements**			
Landry, 2014	Case notes	% partographs completed correctly for c-section deliveries	2
Kihuba, 2014	Register entries	% of 11 selected valid mother and child health data elements recorded	9
Kayode, 2014	Register entries	% of 7 neonatal indicators recorded in DHIS2	96.
Bhattacharya, 2019a (Allen)	Register entries	% of immediate breastfeeding initiation plus keeping baby warm recorded	8
**Completeness averaged across centers**			
Keating, 2019	Register entries	average % APGAR score recorded in Ugandan hospitals during preliminary data quality assessment	9
Keating, 2019	Register entries	average % APGAR score recorded in Ugandan hospitals during baseline assessment	9
Keating, 2019	Register entries	average % APGAR score recorded in Kenyan hospitals during preliminary data quality assessment	9
Keating, 2019	Register entries	average % APGAR score recorded in Kenyan hospitals during baseline assessment	9
Keating, 2019	Register entries	average % birthweight recorded in Ugandan hospitals during preliminary data quality assessment	8
Keating, 2019	Register entries	average % birthweight recorded in Ugandan hospitals during baseline assessment	9
Keating, 2019	Register entries	average % birthweight recorded in Kenyan hospitals during preliminary data quality assessment	8

Keating, 2019	Register entries	average % birthweight recorded in Kenyan hospitals during baseline assessment	9
Keating, 2019	Register entries	average % status of baby at discharge recorded in Ugandan hospitals during preliminary data quality assessment	5
Keating, 2019	Register entries	average % status of baby at discharge recorded in Ugandan hospitals during baseline assessment	6
Keating, 2019	Register entries	average % status of baby at discharge recorded in Kenyan hospitals during preliminary data quality assessment	7
Keating, 2019	Register entries	average % status of baby at discharge recorded in Kenyan hospitals during baseline assessment	9
Keating, 2019	Register entries	average % status of gestational age recorded in Ugandan hospitals during preliminary data quality assessment	5
Keating, 2019	Register entries	average % status of gestational age recorded in Ugandan hospitals during baseline assessment	7
Keating, 2019	Register entries	average % status of gestational age recorded in Kenyan hospitals during preliminary data quality assessment	9
Keating, 2019	Register entries	average % status of gestational age recorded in Kenyan hospitals during baseline assessment	9
**Completeness of aggregate reports**			
Bhattacharya, 2019b (Umar)	Aggregate report	% reporting of early postnatal care in DHIS2 reports	2
Bhattacharya, 2019b (Umar)	Aggregate report	% reporting of birth outcome in DHIS2 reports	4
Bhattacharya, 2019b (Umar)	Aggregate report	% reporting of exclusive breastfeeding 0-6 monhts in DHIS2 reports	2
Bhattacharya, 2019b (Umar)	Aggregate report	% reporting of delivery by skilled birth attendant in DHIS2 reports	2
Bhattacharya, 2019b (Umar)	Aggregate report	% reporting of facility deliveries in DHIS2 reports	5
Bhattacharya, 2019b (Umar)	Aggregate report	% reporting of polio vaccine given at birth in DHIS2 reports	5
Bhattacharya, 2019b (Umar)	Aggregate report	% reporting of BCG vaccine in DHIS2 reports	5
Ndira, 2008	Aggregate report	% reporting of date of submission of reports to district health office	68.
**Timeliness of aggregate reports**			
Ndira, 2008	Aggregate reports	% of regular aggregate reports submitted on time	8

Table S6: Estimates of internal consistency

Author, year	Data source	Measure	Point estimate
**Included in figure 4**			
Kong, 2020	Register entries	Analogue birthweight heaping (% heaped values)	37.1
Kong, 2020	Register entries	Digital birthweight heaping (% heaped values)	17.1
Day, 2020a (Gore)	Register entries	Birthweight heaping (% heaped values in Azimpur, Bangladesh)	17.49
Day, 2020a (Gore)	Register entries	Birthweight heaping (% heaped values in Kushtia, Bangladesh)	58.43
Day, 2020a (Gore)	Register entries	Birthweight heaping (% heaped values in Pokhara, Nepal)	34.09
Day, 2020a (Gore)	Register entries	Birthweight heaping (% heaped values in Temeke, Tanzania)	42.81
Day, 2020a (Gore)	Register entries	Birthweight heaping (% heaped values in Muhimbili, Tanzania)	16.97
Miller, 2020	Register entries	Inconsistent gestational age (% records in Kenya where birthweight for gestational age falls below 3rd/above 97th percentile)	12.3
Miller, 2020	Register entries	Inconsistent gestational age (% records in Uganda where birthweight for gestational age falls below 3rd/above 97th percentile	11.4
Miller, 2020	Register entries	Inconsistent gestational age (% discrepancies between recorded gestational age and gestational age calculated from last menstrual period in Kenya)	70.5
Day, 2020a (Gore)	Register entries	Inconsistent birth outcome (% stillbirths with early breastfeeding initiation in Temeke, Tanzania)	11.5

Day, 2020a (Gore)	Register entries	Inconsistent birth outcome (% stillbirths discharged as "alive" or "well" in Kushtia, Bangladesh)	5.4
Day, 2020a (Gore)	Register entries	Inconsistent birth outcome (% stillbirths discharged as "alive" or "well" in Temeke, Tanzania)	6.6
Day, 2020a (Gore)	Register entries	Inconsistent birth outcome (% stillbirths discharged as "alive" or "well" in Muhimbili, Tanzania)	16.3
Day, 2020a (Gore)	Register entries	Inconsistent birth outcome (% stillbirths discharged as "unwell" in Azimpur, Bangladesh)	96.2
Day, 2020a (Gore)	Register entries	Inconsistent birth outcome (% stillbirths discharged as "unwell" in Kushtia, Bangladesh)	94.6
Kc, 2020	Register entries	Inconsistent birth outcome (% potentially misclassified intrapartum stillbirths in Azimpur, Bangladesh)	46
Miller, 2020	Register entries	Gestational age outliers (% <24 weeks in Kenya)	0.3
Miller, 2020	Register entries	Gestational age outliers (% >42 weeks in Kenya)	0.3
Miller, 2020	Register entries	Gestational age outliers (% <24 weeks in Uganda)	0.7
Miller, 2020	Register entries	Gestational age outliers (% >42 weeks in Uganda)	0.2
Day, 2020a (Gore)	Register entries	Gestational age outliers (% <20 or >44 weeks in Muhimbili, Tanzania)	0.22
Day, 2020a (Gore)	Register entries	Gestational age outliers (% <20 il >44 weeks in Pokhara, Nepal)	0.16
Miller, 2020	Register entries	Birthweight outliers (% <500g in Kenya)	3.8

Miller, 2020	Register entries	Birthweight outliers (% >6000g in Kenya)	0
Miller, 2020	Register entries	Birthweight outliers (% <500g in Uganda)	13.3
Miller, 2020	Register entries	Birthweight outliers (% >6000g in Uganda)	0
Kong, 2020	Register entries	Birthweight outliers (% <350g or >5000g)	0.03
Day, 2020a (Gore)	Register entries	Birthweight outliers (% <350g or >5000g in Temeke, Tanzania)	0.35
Day, 2020a (Gore)	Register entries	Birthweight outliers (% <350g or >5000g in Muhimbili, Tanzania)	1.19
Day, 2020a (Gore)	Register entries	Birthweight outliers (% <350g or >5000g in Azimpur, Bangladesh)	0
Day, 2020a (Gore)	Register entries	Birthweight outliers (% <350g or >5000g in Kushtia, Bangladesh)	0
Day, 2020a (Gore)	Register entries	Birthweight outliers (% <350g or >5000g, in Pokhara, Nepal)	0.01
**Excluded from figure 4**			
**Author, year**	**Data source**	**Measure**	
Miller, 2020	Register	Inconsistent gestational age (median difference in weeks between recorded gestational age and gestational age calculated from last menstrual period)	1.7

Table S7: Estimates of external consistency

**Included in figure 5**

Author, year	Measure	Data source	Comparison	Data element
**Specificity, Register entries**				
Broughton, 2013	Specificity	Register entries	direct observation	Neonatal death (in first hour after birth)
Bhattacharya, 2019a (Allen)	Specificity	Register entries	direct observation	Birth outcome (fresh and macerated stillbirth)
Broughton, 2013	Specificity	Register entries	direct observation	Birth outcome (stillbirth)
Peven, 2021	Specificity	Register entries	direct observation	Birth outcome (stillbirth)
Bhattacharya, 2019a (Allen)	Specificity	Register entries	direct observation	Profession of birth attendant
Broughton, 2013	Specificity	Register entries	direct observation	Asphyxia
Day, 2020b (Rahman)	Specificity	Register entries	direct observation	Bag mask ventilation
Kc, 2021	Specificity	Register entries	direct observation	Bag mask ventilation (among all live births and fresh stillbirths)
Kc, 2021	Specificity	Register entries	direct observation	Bag mask ventilation (among non-crying infants)
Kc, 2021	Specificity	Register entries	direct observation	Bag mask ventilation (among non-breathing infants)
Bhattacharya, 2019a (Allen)	Specificity	Register entries	direct observation	Birthweight (baby weighed at birth)
Bhattacharya, 2019a (Allen)	Specificity	Register entries	direct observation	Birthweight (low birthweight (<2500 g))
Kong, 2020	Specificity	Register entries	direct observation	Birthweight (low birthweight (<2500 g))
Kong, 2020	Specificity	Register entries	direct observation	Birthweight (baby weighed at birth)
Kc, 2021	Specificity	Register entries	direct observation	Stimulation (among all live births and fresh stillbirths)

Zaman, 2021	Specificity	Register entries	direct observation	Cord care (Chlorhexidrine application in all live births, not-recorded an[illegible] no)
Day, 2020b (Rahman)	Specificity	Register entries	direct observation	Early initiation of breastfeeding (within 1 hour of birth)
Tahsina, 2021	Specificity	Register entries	direct observation	Early initiation of breastfeeding
**Specificitiy, Case notes**				
Broughton, 2013	Specificity	Case notes	direct observation	Cord care
Broughton, 2013	Specificity	Case notes	direct observation	Dry and wrap
Broughton, 2013	Specificity	Case notes	direct observation	Early initiation of breastfeeding (within 1 hour of birth)
**Sensitivity, Register entries**				
Broughton, 2013	Sensitivity	Register entries	direct observation	Neonatal death (in first hour after birth)
Bhattacharya, 2019a (Allen)	Sensitivity	Register entries	direct observation	Birth outcome (fresh and macerated stillbirth)
Broughton, 2013	Sensitivity	Register entries	direct observation	Birth outcome (stillbirth)
Peven, 2021	Sensitivity	Register entries	direct observation	Birth outcome (stillbirth)
Bhattacharya, 2019a (Allen)	Sensitivity	Register entries	direct observation	Profession of birth attendant
Broughton, 2013	Sensitivity	Register entries	direct observation	Asphyxia
Day, 2020b (Rahman)	Sensitivity	Register entries	direct observation	KMC initiation
Salim, 2021	Sensitivity	Register entries	direct observation	KMC initiation
Day, 2020b (Rahman)	Sensitivity	Register entries	direct observation	Bag mask ventilation
Kc, 2021	Sensitivity	Register entries	direct observation	Bag mask ventilation (among all live births and fresh stillbirths)
Kc, 2021	Sensitivity	Register entries	direct observation	Bag mask ventilation (among non-crying infants)

Kc, 2021	Sensitivity	Register entries	direct observation	Bag mask ventilation (among non-breathing infants)
Bhattacharya, 2019a (Allen)	Sensitivity	Register entries	direct observation	Birthweight (baby weighed at birth)
Bhattacharya, 2019a (Allen)	Sensitivity	Register entries	direct observation	Birthweight (low birthweight (<2500 g))
Kong, 2020	Sensitivity	Register entries	direct observation	Birthweight (low birthweight (<2500 g))
Kong, 2020	Sensitivity	Register entries	direct observation	Birthweight (baby weighed at birth)
Kc, 2021	Sensitivity	Register entries	direct observation	Stimulation
Zaman, 2021	Sensitivity	Register entries	direct observation	Cord care (Chlorhexidrine application in all live births, not-recorded and no)
Day, 2020b (Rahman)	Sensitivity	Register entries	direct observation	Early initiation of breastfeeding (within 1 hour of birth)
Tahsina, 2021	Sensitivity	Register entries	direct observation	Early initiation of breastfeeding
**Sensitivity, Case notes**				
Broughton, 2013	sensitivity	Case notes	direct observation	Cord care
Broughton, 2013	sensitivity	Case notes	direct observation	Dry and wrap
Broughton, 2013	sensitivity	Case notes	direct observation	Early initiation of breastfeeding (within 1 hour of birth)
**Percent agreement, Register entries**				
Broughton, 2013	% agreement	Register entries	direct observation	Neonatal death (in first hour after birth)
Bhattacharya, 2019a (Allen)	% agreement	Register entries	direct observation	Gestational age (preterm birth)
Bhattacharya, 2019a (Allen)	% agreement	Register entries	direct observation	Birth outcome (fresh and macerated stillbirth)
Broughton, 2013	% agreement	Register entries	direct observation	Birth outcome (stillbirth)
Peven, 2021	% agreement	Register entries	direct observation	Birth outcome (stillbirth)

Bhattacharya, 2019a (Allen)	% agreement	Register entries	direct observation	Profession of birth attendant
Broughton, 2013	% agreement	Register entries	direct observation	Asphyxia
Day, 2020b (Rahman)	% agreement	Register entries	direct observation	KMC initiation
Salim, 2021	% agreement	Register entries	direct observation	KMC initiation
Day, 2020b (Rahman)	% agreement	Register entries	direct observation	Bag mask ventilation
Kc, 2021	% agreement	Register entries	direct observation	Bag mask ventilation (among all live births and fresh stillbirths)
Kc, 2021	% agreement	Register entries	direct observation	Bag mask ventilation (among non-crying infants)
Kc, 2021	% agreement	Register entries	direct observation	Bag mask ventilation (among non-breathing infants)
Bhattacharya, 2019a (Allen)	% agreement	Register entries	direct observation	Birthweight (baby weighed at birth)
Bhattacharya, 2019a (Allen)	% agreement	Register entries	direct observation	Birthweight (low birthweight (<2500 g))
Kong, 2020	% agreement	Register entries	direct observation	Birthweight (low birthweight (<2500 g))
Kong, 2020	% agreement	Register entries	direct observation	Birthweight (baby weighed at birth)
Kc, 2021	% agreement	Register entries	direct observation	Stimulation
Duffy, 2009	% agreement	Register entries	direct observation	Mode of delivery (vacuum delivery)
Duffy, 2009	% agreement	Register entries	direct observation	Mode of delivery (c-section)
Zaman, 2021	% agreement	Register entries	direct observation	Cord care (Chlorhexidrine application in all live births, not-recorded and no)

Zaman, 2021	% agreement	Register entries	direct observation	Cord care (Chlorhexidrine application in vaginal births, not-recorded an no)
Zaman, 2021	% agreement	Register entries	direct observation	Cord care (Chlorhexidrine application in c-section births, not-recorded, no)
Zaman, 2021	% agreement	Register entries	direct observation	Cord care (Chlorhexidrine application in all live births, excluding not-rec
Zaman, 2021	% agreement	Register entries	direct observation	Cord care (Chlorhexidrine application in vaginal births, excluding not-re
Zaman, 2021	% agreement	Register entries	direct observation	Cord care (Chlorhexidrine application in c-section births, excluding not- readable)
Day, 2020b (Rahman)	% agreement	Register entries	direct observation	Early initiation of breastfeeding (within 1 hour of birth)
Tahsina, 2021	% agreement	Register entries	direct observation	Early initiation of breastfeeding
**Percent agreement, Case notes**				
Broughton, 2013	% agreement	Case notes	direct observation	Cord care
Broughton, 2013	% agreement	Case notes	direct observation	Dry and wrap
Broughton, 2013	% agreement	Case notes	direct observation	Early initiation of breastfeeding (within 1 hour of birth)
**Excluded from figure 5**				

**Author, year**	**Measure**	**Data source**	**Comparison**	**Data element**
**Measure other than specificity, sensitivity, and percent agreement in case notes or register entries**				
Bhattacharya, 2019a (Allen)	AUC	Register entries	direct observation	Profession of birth attendant
Bhattacharya, 2019a (Allen)	AUC	Register entries	direct observation	Essential newborn care (immediate breastfeeding and keeping baby wa
Bhattacharya, 2019a (Allen)	AUC	Register entries	direct observation	Birthweight
Bhattacharya, 2019a (Allen)	AUC	Register entries	direct observation	Gestational age
Bhattacharya, 2019a (Allen)	AUC	Register entries	direct observation	Birth outcome

Bhattacharya, 2019a (Allen)	Inflation factor	Register entries	direct observation	Profession of birth attendant
Bhattacharya, 2019a (Allen)	Inflation factor	Register entries	direct observation	Essential newborn care (immediate breastfeeding and keeping baby wa
Bhattacharya, 2019a (Allen)	Inflation factor	Register entries	direct observation	Birthweight
Bhattacharya, 2019a (Allen)	Inflation factor	Register entries	direct observation	Gestational age
Bhattacharya, 2019a (Allen)	Inflation factor	Register entries	direct observation	Birth outcome
Broughton, 2013	AUC	Register entries	direct observation	Asphyxia
Broughton, 2013	AUC	Register entries	direct observation	Stillbirth
Broughton, 2013	AUC	Register entries	direct observation	Neonatal death in first hour after birth
Broughton, 2013	AUC	Case notes	direct observation	Breastfeeding initiation in first hour after birth
Broughton, 2013	AUC	Case notes	direct observation	Dry and wrap newborn
Broughton, 2013	AUC	Case notes	direct observation	Cord care
Broughton, 2013	% positive discordance	Register entries	direct observation	Asphyxia
Broughton, 2013	% positive discordance	Register entries	direct observation	Stillbirth
Broughton, 2013	% positive discordance	Case notes	direct observation	Breastfeeding initiation in first hour after birth
Broughton, 2013	% positive discordance	Case notes	direct observation	Dry and wrap newborn
Broughton, 2013	% positive discordance	Case notes	direct observation	Cord care
Broughton, 2013	% positive discordance	Register entries	direct observation	Neonatal death
Zaman, 2021	Validity ratio	Register entries	direct observation	Cord care

Zaman, 2021	Validity ratio	Register entries	direct observation	Cord care
Zaman, 2021	Validity ratio	Register entries	direct observation	Cord care
Kong, 2020	Ratio	Register entries	direct observation	Low birthweight
Kong, 2020	Ratio	Register entries	direct observation	Normal birthweight
Day, 2020b (Rahman)	Validity ratio	Register entries	direct observation	Bag mask ventilation
Day, 2020b (Rahman)	Validity ratio	Register entries	direct observation	Initiation of breastfeeding in hour after birth
Day, 2020b (Rahman)	Validity ratio	Register entries	direct observation	KMC
**Composite newborn data element**				
Bhattacharya, 2019a (Allen)	specificity	Register entries	direct observation	Essential newborn care (immediate breastfeeding initiation and keepin
Bhattacharya, 2019a (Allen)	sensitivity	Register entries	direct observation	Essential newborn care (immediate breastfeeding initiation and keepin
Bhattacharya, 2019a (Allen)	% agreement	Register entries	direct observation	Essential newborn care (immediate breastfeeding initiation and keepin
**Comparison with aggregate data**				
Plotkin, 2018	specificity	Register entries	death audit	Fresh stillbirth
Plotkin, 2018	sensitivity	Register entries	death audit	Fresh stillbirth
Plotkin, 2018	specificity	Register entries	death audit	Macerated stillbirth
Plotkin, 2018	sensitivity	Register entries	death audit	Macerated stillbirth
Plotkin, 2018	specificity	Register entries	death audit	Neonatal death

Plotkin, 2018	sensitivity	Register entries	death audit	Neonatal death
Sychareun, 2014	% agreement	Register entries	MCH reports, HMIS	Early postnatal care
Sychareun, 2014	% agreement	Register entries	MCH reports, HMIS	Skilled birth attendant
Ndira, 2008	% agreement	Maternal recall	district health center reports	Stillbirth
Mony, 2015	sensitivity	Register entries	capture-recapture	Early neonatal death
Mony, 2015	sensitivity	Register entries	capture-recapture	Perinatal death
Bhattacharya, 2020	ICC	Register entries	DHIS2 monthly reports	Early postpartum care (within 3 days of birth)
Bhattacharya, 2020	ICC	Register entries	DHIS2 monthly reports	Facility deliveries
Nichol, 2016	Correlation	Register entries	monthly reports	Nevirapine prophylaxis less than 72 hours after delivery
Phillips, 2019	Difference	Facility records	Government administrative data	Mean frequency of deliveries
Phillips, 2019	Difference	Facility records	Government administrative data	Mean frequency of c-section
Mate, 2009	Average % deviation	Register entries	DHIS monthly reports	Nevirapine dose to baby born to woman with HIV
Mate, 2009	% within 10%	Register entries	DHIS monthly reports	Nevirapine dose to baby born to woman with HIV

Table S8: Additional findings on other measures of data quality

**Author, year**	**Data source**	**Measure of data quality**	**Point estima**
**In case notes and register entries**			
Lambo, 2011	Registers	% of private hospitals with birth registers	

Lambo, 2011	Registers	% of private hospitals with inpatient registers
Lambo, 2011	Registers	% of private hospitals with outpatient registers
Lambo, 2011	Registers	% of public hospitals with birth registers
Lambo, 2011	Registers	% of public hospitals with inpatient registers
Lambo, 2011	Registers	% of public hospitals with outpatient registers
Duffy, 2009	Register entries	% of observed births recorded in delivery room logbook
Landry, 2014	Case notes	% maternal files found for c-section deliveries
Day, 2020b (Rahman)	Register entries	% illegible initiated breastfeeding within 1 hour of birth
Day, 2020b (Rahman)	Register entries	% illegible bag mask ventilation
Day, 2020b (Rahman)	Register entries	% illegibile KMC
Kong, 2020	Register entries	% illegible birthweight
Tahsina, 2021	Register entries	% illegible early initiation of breastfeeding
Salim, 2021	Register entries	% illegible KMC
Kong, 2020	Register entries	% illegible birthweight
Tahsina, 2021	Register entries	% illegible early initiation of breastfeeding
Salim, 2021	Register entries	% illegible KMC
Kc, 2021	Register entries	% illegible stimulation among all live births and stillbirths
Kc, 2021	Register entries	% illegible BMV among all live births and stillbirths
Kc, 2021	Register entries	% illegible BMV among non-crying live births
Kc, 2021	Register entries	% illegible BMV among non-breathing live births
Peven, 2021	Register entries	% illegible birth outcome
Zaman, 2021	Register entries	% illegible chlorhexadrine application for all live births
Zaman, 2021	Register entries	% illegible chlorhexadrine application for vaginal births
Zaman, 2021	Register entries	% illegible chlorhexadrine application for c-section births
Chiba, 2012	Register entries	% illegible mode of delivery data at Bondo hospital
Chiba, 2012	Register entries	% illegible date of delivery data at Siaya hospital
Chiba, 2012	Register entries	% illegible mode of delivery data at Siaya hospital
Chiba, 2012	Register entries	% illegible Apgar score data at Siaya hospital
Chiba, 2012	Register entries	% illegible sex data at Siaya hospital
Nyamtema, 2008	Case notes	% partograms not completed according to standardized protocols

Chiba, 2012	Register entries	% incorrectly coded infant feeding data at Bondo hospital
Chiba, 2012	Register entries	% incorrectly coded infant feeding data at Siaya hospital
Chiba, 2012	Register entries	% incorrectly coded mode of delivery data at Bondo hospital
Chiba, 2012	Register entries	% incorrectly coded mode of delivery data at Siaya hospital
Chiba, 2012	Register entries	% incorrectly coded sex data at Bondo hospital
Chiba, 2012	Register entries	% incorrectly coded sex data at Siaya hospital
Hazard, 2017	Case notes	% newborn medical records of high quality based on Gates 13 Grand Challenges in Health study gold standard criteria
**In aggregate reports**		
Bhattacharya, 2019b (Umar)	Aggregate reports	mean of % of annual reports submitted by facilities to DHIS2 across 10 facilities
Bhattacharya, 2019b (Umar)	Aggregate reports	mean number of annual reports submitted by facilities to DHIS across 10 facilities
Bhattacharya, 2019b (Umar)	Aggregate reports	mean % of annual reports submitted on time by facilities to DHIS2 across 10 facilities
Ndira, 2008	Aggregate reports	% regular reports from MCH unit found in district health office

Table S9: Quality of methodology assessment

		**Quality of methodology criteria***					
**Author, year**	**Quality score**	**1**	**2**	**3**	**4**	**5**	**6**
Keating, 2019	19	1	1	1	1	1	1
Day, 2020a	18	1	1	1	1	1	1
Day, 2020b	18	1	1	1	1	1	1
Kayode, 2014	18	1	1	1	1	1	1
KC, 2021	18	1	1	1	1	1	1
Kihuba, 2014	18	1	1	1	1	1	1
Kong, 2020	18	1	1	1	1	1	1
Lambo, 2011	18	1	1	1	1	1	1
Peven, 2021	18	1	1	1	1	1	1

Plotkin, 2018	18	1	1	1	1	1	1
Salim, 2021	18	1	1	1	1	1	1
Tahsina, 2021	18	1	1	1	1	1	1
Zaman, 2021	18	1	1	1	1	1	1
Bhattacharya, 2019a	17	1	1	1	1	1	1
Broughton, 2013	17	1	1	1	1	1	1
Chiba, 2012	17	1	1	1	1	1	1
Hazard, 2017	17	1	1	1	1	1	1
Landry, 2014	17	1	1	1	1	1	1
Miller, 2020	17	1	1	1	1	1	1
Phillips, 2019	17	1	1	1	1	1	1
Rahman, 2021	17	1	1	1	1	1	1
Sharma, 2016	17	1	1	1	1	1	0
Sychareun, 2014	17	1	1	1	1	1	1
Bhattacharya, 2019b	16	1	1	1	1	1	1
Bhattacharya, 2020	16	1	1	0	1	1	1
Gebrehiwot, 2020	16	1	1	0	1	1	1
Mony, 2015	16	1	1	1	1	1	1
Ndira, 2008	16	1	1	1	1	1	1
Fawole, 2007	15	1	1	0	1	1	1
KC, 2020	15	1	1	0	1	1	1
Mate, 2009	15	1	1	1	1	1	1
Nyamtema, 2008	15	1	1	1	1	1	1
Duffy, 2009	14	1	1	0	1	1	1
Nicol, 2016	14	1	1	0	1	1	1

* Quality of methodology criteria based on Critical appraisal tool to assess the quality of cross-sectional studies (AXIS) with minor adaptations; 1-cl
population, 6- adequate selection process for sample, 7- attention to non -recorded records/indicators/data elements, 8- appropriate measureme
described, 13- information provided on non-recorded data, 14- results internally consistent, 15- results reflect methods described, 16- discussion/
